# Peptide‐Based Biomaterials as a Promising Tool for Cancer Radiotherapy

**DOI:** 10.1002/advs.202501775

**Published:** 2025-07-16

**Authors:** Qian Wang, Xinhui Chu, Jianfeng Liu

**Affiliations:** ^1^ State Key Laboratory of Advanced Medical Materials and Devices Tianjin Key Laboratory of Radiation Medicine and Molecular Nuclear Medicine Key Laboratory of Radiopharmacokinetics for Innovative Drugs Tianjin Institutes of Health Science Institute of Radiation Medicine Chinese Academy of Medical Sciences & Peking Union Medical College Tianjin 300192 P. R. China

**Keywords:** peptide‐based biomaterials, radiopharmaceuticals, radiosensitizers, radiotherapy

## Abstract

Radiotherapy (RT) is a mainstay therapeutic strategy for cancer; however, increasing radiation damage to tumor tissues while reducing the side effects on healthy tissues remains a great challenge. To address this issue, the use of biomaterials has proven promising. Recently, attention has been paid to peptide‐based biomaterials as platforms for enhancing radiotherapeutic efficacy. This review explores peptide‐based biomaterial‐mediated tumor RT, including radiosensitizers and radiopharmaceuticals, with the aim of introducing emerging radiosensitive methods and RT strategies using radiopharmaceuticals. The advantages of peptide‐based biomaterials, including controllable synthesis, good biocompatibility, targeting functions, and self‐assembly performance, are introduced. These parameters must be considered in the rational design and optimization of peptide‐based RT strategies. Peptide‐based radiosensitizers are generally divided into three categories: peptides as direct radiosensitizers, peptides as carriers of radiosensitizers, and targeted‐peptide‐modified radiosensitizers. Peptide‐based radiopharmaceuticals are known as peptide‐radionuclide conjugates (PRCs) and are categorized according to their peptide targets. Details of PRCs used in clinical studies and US Food and Drug Administration‐approved PRCs are also presented. Finally, challenges in the clinical translation of peptide‐based biomaterials as RT tools are highlighted.

## Graphical abstract

This review provides a systematic overview of peptide‐based biomaterials in tumor radiotherapy, covering their roles as both radiosensitizers and radiopharmaceuticals. Peptide‐based radiosensitizers are generally divided into three categories: peptides as direct radiosensitizers, peptides as carriers of radiosensitizers, and targeted‐peptide‐modified radiosensitizers. Peptide‐based radiopharmaceuticals are known as peptide‐radionuclide conjugates and are categorized according to their peptide targets.

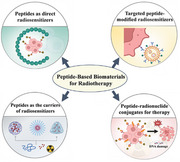

## 1 Introduction

Cancer is considered one of the most challenging diseases globally, as it seriously threatens human health. According to the World Health Organization, cancer‐related deaths are projected to increase by 45% between 2007 and 2030.^[^ ^1^ ^]^ Several therapeutic strategies have been developed for cancer treatment, including surgery, chemotherapy, and radiotherapy (RT).^[^ ^2^ ^]^ RT is widely used in clinical practice, with ≈60% of patients with cancer receiving RT in the United States.^[^ ^3^ ^]^ RT uses high‐energy ionizing radiation (IR) to damage DNA and other cellular components, thereby inducing apoptosis or necrosis.^[^ ^1^, ^2^, ^4^ ^]^ Compared with other treatments, RT has superior tumor tissue penetration because the X‐ or γ‐rays penetrate deeper into tumor tissues.^[^ ^5^ ^]^ However, long‐term treatment can have serious drawbacks, such as the obstruction of healthy tissue and radioresistance.^[^ ^4^ ^]^ To overcome radioresistance and reduce the damage to normal tissues caused by RT, it is necessary to develop innovative RT strategies.

Tumor radioresistance is polymodal and is associated with the adaptive evolution of tumor cells, including an altered cell cycle, modified metabolism, repopulation by cancer stem cells (CSCs), enhanced DNA repair, angiogenesis, vasculogenesis, and hypoxia.^[^ ^6^ ^]^ Despite the complex mechanisms that generate radioresistance, they provide valid targets for designing radiosensitization strategies. One way to integrate these targets and overcome radioresistance is by introducing radiosensitizers.^[^ ^7^ ^]^ Radiosensitizers can increase radiation energy deposition at the tumor site and convert the radioresistant signal into a radiosensitive signal, thereby reducing the radiation dose and RT‐induced damage to normal tissue.^[^ ^3^, ^8^ ^]^ In addition, the development of radiopharmaceuticals for tumor theranostics has increased in recent years.^[^ ^9^ ^]^ Radiotheranostics combines molecular imaging with targeted radionuclide therapy.^[^ ^10^ ^]^ Radiotherapy using radiopharmaceuticals has the potential to target and eliminate tumor cells with minimal adverse effects, because it uses internal irradiation, unlike other traditional systemic therapies.^[^ ^11^ ^]^

With the support of advanced technologies, various biomaterials have been designed and explored to improve the efficacy of tumor therapy.^[^ ^12^ ^]^ The properties of the biomaterials used in tumor therapy can be attributed to two factors. The physicochemical properties of biomaterials, such as assembly performance, surface modifications, and drug‐carrying capacity, must be optimized.^[^ ^13^ ^]^ However, biomaterial safety remains a critical issue. Despite encouraging advancements in biomaterials, including polymers,^[^ ^14^ ^]^ liposomes,^[^ ^15^ ^]^ inorganic metals,^[^ ^16^ ^]^ and albumin,^[^ ^17^ ^]^ personalized and intelligent biomaterials still have room for improvement. Peptide‐based biomaterials are a class of functional biomaterials that consist of peptides or peptide derivatives. They have attracted increasing attention because of their properties, including biocompatibility, easy and economical synthesis, modifiability, and enhanced permeability and retention (EPR) effects in tumor tissues after self‐assembly into nanostructures, and because of their unique advantages, including flexible targeting, sensitive signaling responsiveness, and self‐assembly properties with controllable morphology.^[^ ^16b^ ^]^ Amino acids are the main components of peptide‐based biomaterials.^[^ ^18^ ^]^ The type and arrangement of amino acids endow peptide‐based biomaterials with diverse properties. Some peptides, such as endorphins^[^ ^19^ ^]^ or defensins,^[^ ^20^ ^]^ can act on their own, whereas others, such as GFFY, can be used as drug carriers in combination with drugs^[^ ^21^ ^]^ or fluorescent molecules^[^ ^22^ ^]^ for disease theranostics. Peptides can function by themselves or can integrate with other functional groups.^[^ ^23^ ^]^ This design flexibility makes them star molecules in drug development and materials science.

**Figure 1:**
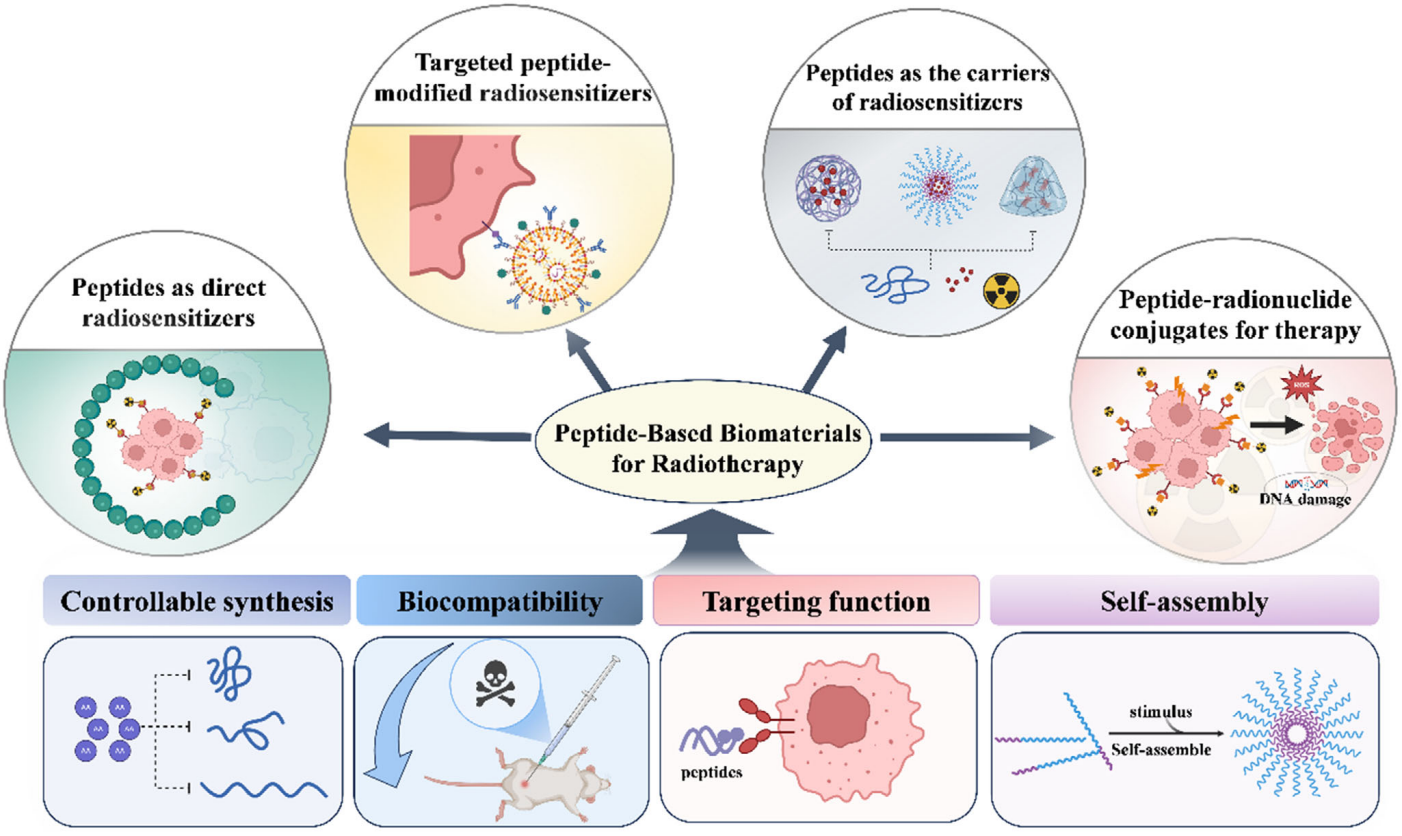
The overview of peptide‐based biomaterials as a promising tool for RT.

Radiosensitizers and radiopharmaceuticals are candidates for innovative RT strategies. The former is an “auxiliary tool” for RT that enhances the tumor's response to radiation through chemical or biological means, and the latter is an “active weapon” that directly uses radioactive isotopes to kill tumor cells or produce images. Radiopharmaceuticals have expanded globally and have been successfully used clinically.^[^ ^24^ ^]^ However, the radiosensitizers and radiopharmaceuticals used to treat tumors depend on effective delivery platforms. Peptide‐based biomaterials may serve as a new class of precision medicines or drug carriers for clinical cancer treatment, and can contribute to the development of radiosensitizers and radiopharmaceuticals. To date, several reviews have been published on peptides or radiosensitizers; however, few have linked peptide‐based biomaterials to RT. Therefore, in this review, we systematically summarize advanced research on peptide‐based biomaterials for RT (**Figure** **1**). We begin by describing the advantages of peptide‐based biomaterials, such as controllable synthesis, biocompatibility, targeting functions, and self‐assembly performance. We further discuss three types of peptide‐based radiosensitizers: peptides as direct radiosensitizers, peptides as carriers of radiosensitizers, and targeted‐peptide‐modified radiosensitizers. Finally, we sum up peptide‐radionuclide conjugates (PRCs) in terms of different targets. This review will help researchers understand the latest advances in peptide‐based RT strategies, provide the necessary foundation and considerations for designing peptide‐based RT strategies, and facilitate the clinical translation of peptide‐based biomaterials for RT.

## 2 The Advantages of Peptide‐Based Biomaterials

Peptides are composed of varying numbers of amino acids (natural and unnatural), which endow them with unique physicochemical properties. Because of their relatively small molecular weight, they are very different from proteins in terms of their structural complexity and design flexibility, despite having similar biological activities. Peptide‐based biomaterials have garnered significant attention because of their outstanding characteristics in biomedicine, tissue engineering, and bioimaging modalities.^[^ ^25^ ^]^ This section describes the critical biological advantages of peptide‐based biomaterials, including their controllable synthesis, biocompatibility, targeting function, and self‐assembly performance (**Table** **1**). These characteristics provide a strong foundation for their application in RT.

**Table 1:** The key advantages of peptide‐based biomaterials.

Category	Advantages in detail	References
Controllable synthesis	Diversity of synthesis methods (direct extraction, chemosynthesis, biosynthesis), diversity of structures	^[^ ^26^, ^27^, ^38^, ^49^ ^]^
Biocompatibility	Low immunogenicity, controllable degradability, rapid clearance	[56, 57, 58]
Targeting function	Multi‐targeting capabilities (tumor cells, immune cells, cancer‐associated fibroblasts, tumor vascular, and others)	[61, 63, 69, 70]
Self‐assembly	Self‐assembly triggered by enzymes, pH, redox reaction, and other factors	[77, 80, 85, 92]

### 2.1 Controllable Synthesis

#### 2.1.1 Diversity of Synthesis Methods

##### Direct Extraction

Natural peptides have been demonstrated to be pivotal sources for novel drugs. They can be extracted from animals, insects, plants, and microorganisms,^[^ ^26^ ^]^ and released by microbial fermentation, proteolysis, and enzymatic hydrolysis.^[^ ^27^ ^]^ For example, skin peptides extracted from frogs exhibit broad‐spectrum antibacterial and antifungal activities,^[^ ^28^ ^]^ whereas the RGVKGPR oligopeptide derived from the enzymatic hydrolysates of the Chinese three‐striped box turtle (*Cuora trifasciata*) has antitumor activity.^[^ ^29^ ^]^ Moreover, a bioactive peptide extracted from traditional Chinese medicine, kangfuxin, can be used to treat chronic atrophic gastritis.^[^ ^30^ ^]^ More than 3000 natural antimicrobial peptides have been reported.^[^ ^31^ ^]^ Hao et al. coupled self‐assembled RADA16 with a natural antimicrobial peptide to develop a multifunctional composite hydrogel that effectively promotes wound healing.^[^ ^32^ ^]^ However, searching for active peptides among the numerous organisms in the vast world requires considerable effort, and their poor stability and immunogenicity restrict their applications in vivo.^[^ ^33^ ^]^

##### Chemosynthesis

**Figure 2:**
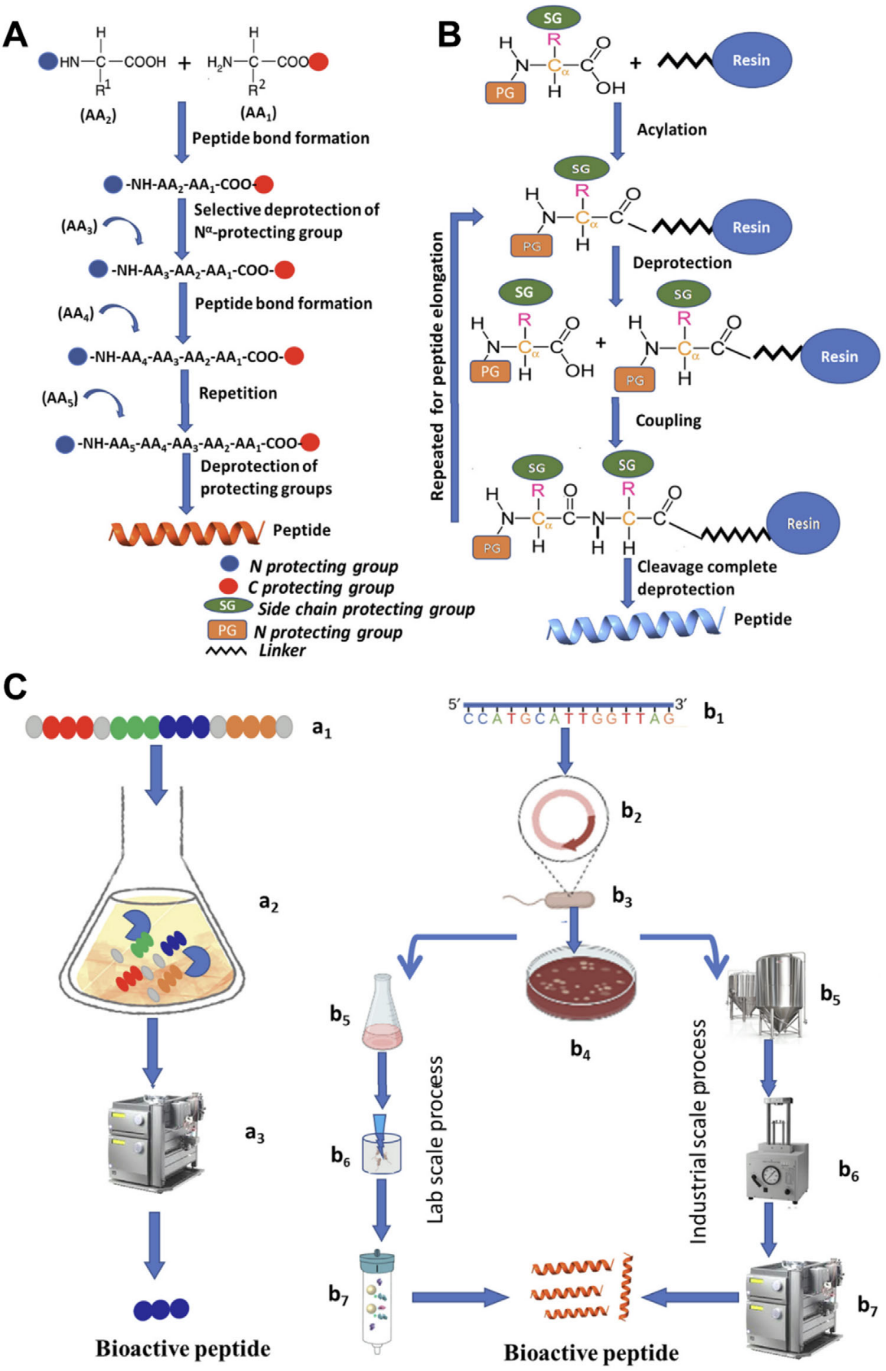
Schematic illustration of peptide synthesis. The process of A) SUPPS and B) SPPS. C) Biosynthesis includes enzymatic hydrolysis method (left) and RDT method (right). Reproduced with permission.^[^ ^38^ ^]^ Copyright 2023, Elsevier Masson SAS.

The chemosynthesis of peptides mainly involves solution‐phase peptide synthesis (SUPPS) and solid‐phase peptide synthesis (SPPS). SUPPS (**Figure** **2**A) was the first method developed for peptide synthesis and is still used today.^[^ ^34^ ^]^ It can be applied under a wide range of experimental conditions, such as catalytic hydrogenation and alkaline hydrolysis, making it suitable for the synthesis of typical peptide structures. However, as the synthesis process occurs in solution, the removal of unreacted raw materials and byproducts is necessary after each peptide connection. SPPS (Figure 2B) is an advanced version of SUPPS and is used to prepare most currently marketed peptide drugs, such as leuprorelin and oxytocin.^[^ ^35^ ^]^ This method requires a solid support as a substrate, such as a polystyrene resin, to bind and immobilize the synthesized peptide. It is fundamentally based on the same principles and includes the following procedures: peptide and amino acid protection with appropriate protecting groups, gradual elongation of the peptide chain, deprotection, and purification.^[^ ^36^ ^]^ Unlike SUPPS, unreacted reagents and byproducts can be easily removed by washing during SPPS.^[^ ^37^ ^]^

##### Biosynthesis

As presented in Figure 2C, peptide biosynthesis can be achieved using enzymatic hydrolysis and recombinant DNA technology (RDT). Compared to chemical synthesis, they have the advantages of low cost, high specificity, and low levels of contamination, and do not require hazardous organic solvents or toxic chemicals.^[^ ^39^ ^]^ Therefore, peptide biosynthesis is widely used for peptide manufacturing.^[^ ^40^ ^]^ Enzymatic hydrolysis involves the hydrolysis of proteins in plants and animals under mild conditions (controlled hydrolysis time, pH, temperature, substrate concentration, and enzymatic activity).^[^ ^41^ ^]^ The anti‐freezing peptide GLLGPLGPRGLL, the antioxidant peptides KALNEINQF and GQGAKDMWR, and the anti‐hypertensive peptide AYFYPEL are obtained through enzyme‐triggered hydrolysis.^[^ ^42^ ^]^ RDT is a feasible method for peptide production because of the ease of peptide sequence optimization for drug conjugation and chemical modification.^[^ ^43^ ^]^ The production process is based on gene expression and cloning in the constructed system, while bacteria are the most commonly used gene expression system.^[^ ^44^ ^]^ RDT involves the following steps: target peptide selection, gene cloning, expression vector construction, transformation, extraction, and purification.^[^ ^38^ ^]^

Controllable synthesis is a prerequisite for the design of radiosensitizers and radiopharmaceuticals. Natural peptide‐based biomaterials have therapeutic potential for different diseases, but have relatively few applications in RT. SPPS can achieve automated production,^[^ ^45^ ^]^ high‐throughput screening, and reduced side reactions, and is often used for the production of short peptides.^[^ ^38^ ^]^ Enzymatic hydrolysis is relatively simple and can be optimized to produce specific peptides, whereas RDT can efficiently and cost‐effectively produce peptides for large‐scale production.^[^ ^46^ ^]^ Overall, the preparation method for RT‐related peptide‐based biomaterials can be flexibly selected according to actual needs.

#### 2.1.2 Diversity of Structures

**Figure 3:**
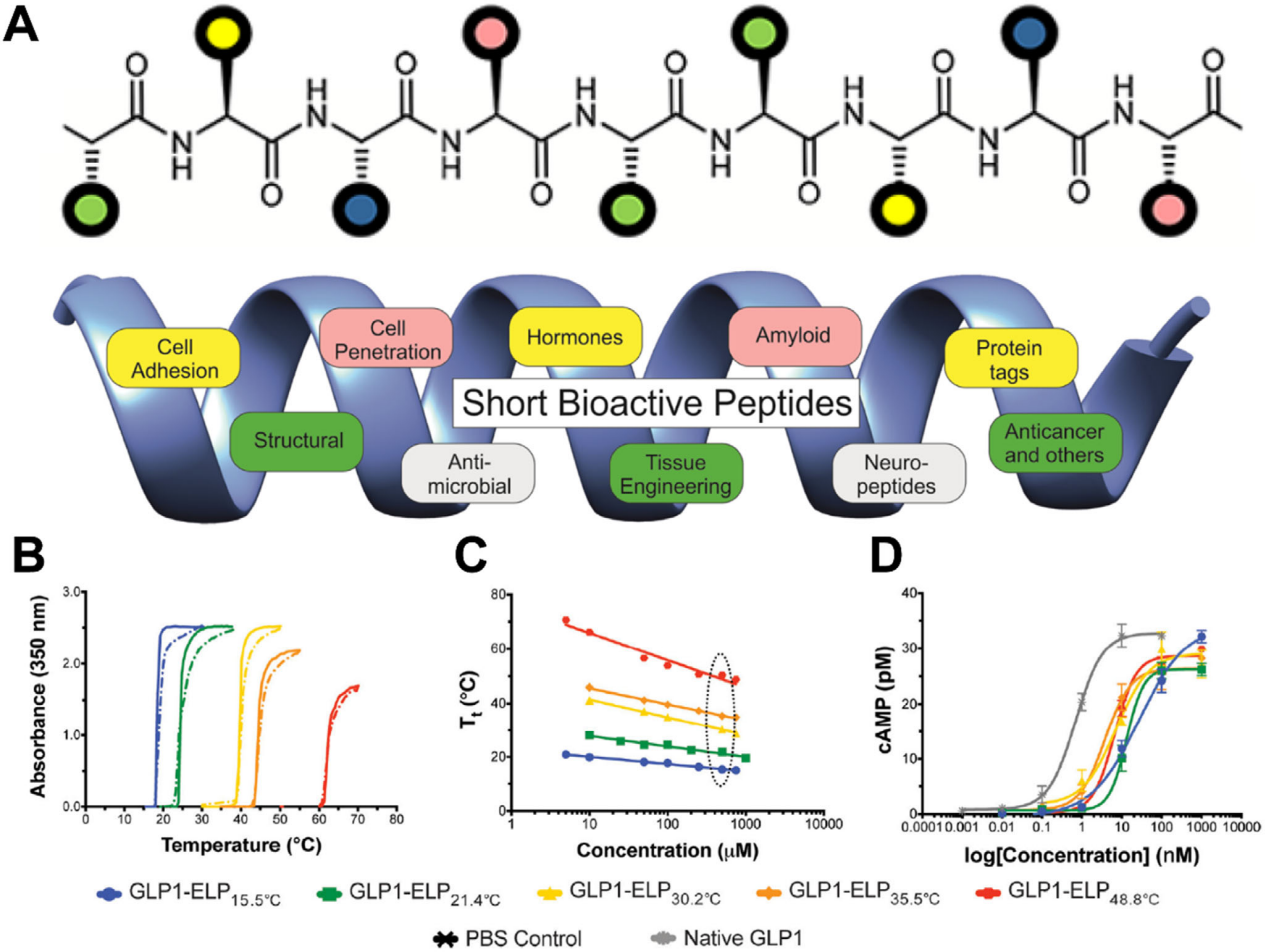
A) The structural diversity of peptides determines their functional diversity. Reproduced with permission.^[^ ^49^ ^]^ Copyright 2017, American Chemical Society B) Reversible phase behavior of the fusions. C) Tt's concentration dependence of the fusions. D) cAMP activity of the fusions. Reproduced with permission.^[^ ^53^ ^]^ Copyright 2017, Springer Nature.

The abundance of synthetic methods has enabled the identification of peptides with structural diversity (**Figure** **3**A),^[^ ^47^ ^]^ such as radiosensitive peptides,^[^ ^48^ ^]^ cell‐penetrating peptides, tumor‐homing peptides, antimicrobial peptides, and growth factors.^[^ ^49^ ^]^ Based on their different structures, some peptide molecules have a direct mode of action, whereas others act as vectors or adjuvants. Peptides composed of natural amino acids (mostly in the L configuration) are susceptible to degradation by serum proteases, resulting in poor stability and efficacy. Various strategies have been explored to prevent this constraint through chemical modifications, including cyclization of peptides; the substitution of α‐amino acids with D‐amino acids or α, α‐disubstituted amino acids; the use of unnatural amino acids that are uncleavable by endogenous proteases; and blocking access to the N‐ and C‐ terminal fragments.^[^ ^50^ ^]^ Moreover, the changes in the structure of peptides affect their immunogenicity. For example, adjusting the assembly structure of peptides by introducing proline and aromatic residues can mimic the natural epitope conformation; the optimization of the key anchoring amino acid residues can improve the affinity of the peptide for MHC class 1 molecules;^[^ ^51^ ^]^ and long‐chain peptides compared to short‐chain peptides have the benefit of inducing an effective CD8^+^ T cell response, since long chains are more easily presented by DC.^[^ ^52^ ^]^

The structures of peptides also endow them with adjustable morphological transformations and specific functions. Elastin‐like polypeptides (ELPs) consist of repeats of a Val‐Pro‐Gly‐Xaa‐Gly motif, where Xaa is any amino acid except Pro. These peptides can undergo temperature phase transitions at any desired temperature (T_t_) between 0 and 100 °C by altering the sequence level.^[^ ^54^ ^]^ Chilkoti et al. constructed a series of fusions between glucagon‐like peptide‐1 (GLP1) and ELPs with different T_t_ values (Figure 3B and C).^[^ ^53^ ^]^ As the T_t_ value changed, the fusion peptides exhibited different cyclic adenosine monophosphate activities (Figure 3D). Moreover, peptides triggered by the proline amide bond isomerization can undergo morphological transformation between nanofibrils and nanoparticles under different pH conditions, thus facilitating tumor accumulation and retention at tumor sites.^[^ ^55^ ^]^ The structure and function of peptide‐based biomaterials are closely related and are the key issues to be considered in RT applications.

### 2.2 Biocompatibility

Because of the high structural similarity between peptides and human proteins, peptide‐based biomaterials exhibit good biocompatibility and controllable degradability in the body.^[^ ^56^ ^]^ Their good biocompatibility provides them with broad application prospects in drug delivery, tissue engineering, and regenerative medicine.

Their short half‐life and rapid in vivo clearance of peptides endow them with good biocompatibility. Hydrophilic peptides with MW ≈2–25 kDa are susceptible to rapid filtration through the glomeruli of the kidney, which have an 8 nm pore size. Because peptides are not easily reabsorbed through the renal tubules, they frequently have high renal clearance.^[^ ^57^ ^]^ In contrast, peptides are cleared by the proteasome and liver‐metabolism‐mediated endocytosis and degradation (e.g., cytochrome P450 mediated oxidation), which accounts for a fraction of the peptide degradation process.^[^ ^58^ ^]^ Correspondingly, because of the good biocompatibility of peptides, drug–drug interactions and non‐mechanistic‐based toxicology are rarely observed. Moreover, the metabolic products of peptides are amino acids that have minimal toxicity under physiological conditions, and peptides are generally unable to pass through the blood–brain barrier due to size limitations, which precludes their toxicology to the central nervous system.^[^ ^59^ ^]^ The biocompatibility of peptide‐based biomaterials ensures the safety of their in vivo application and lays a solid foundation for their clinical translation.

### 2.3 Targeting Function

#### 2.3.1 Tumor Cell Targeting

**Figure 4:**
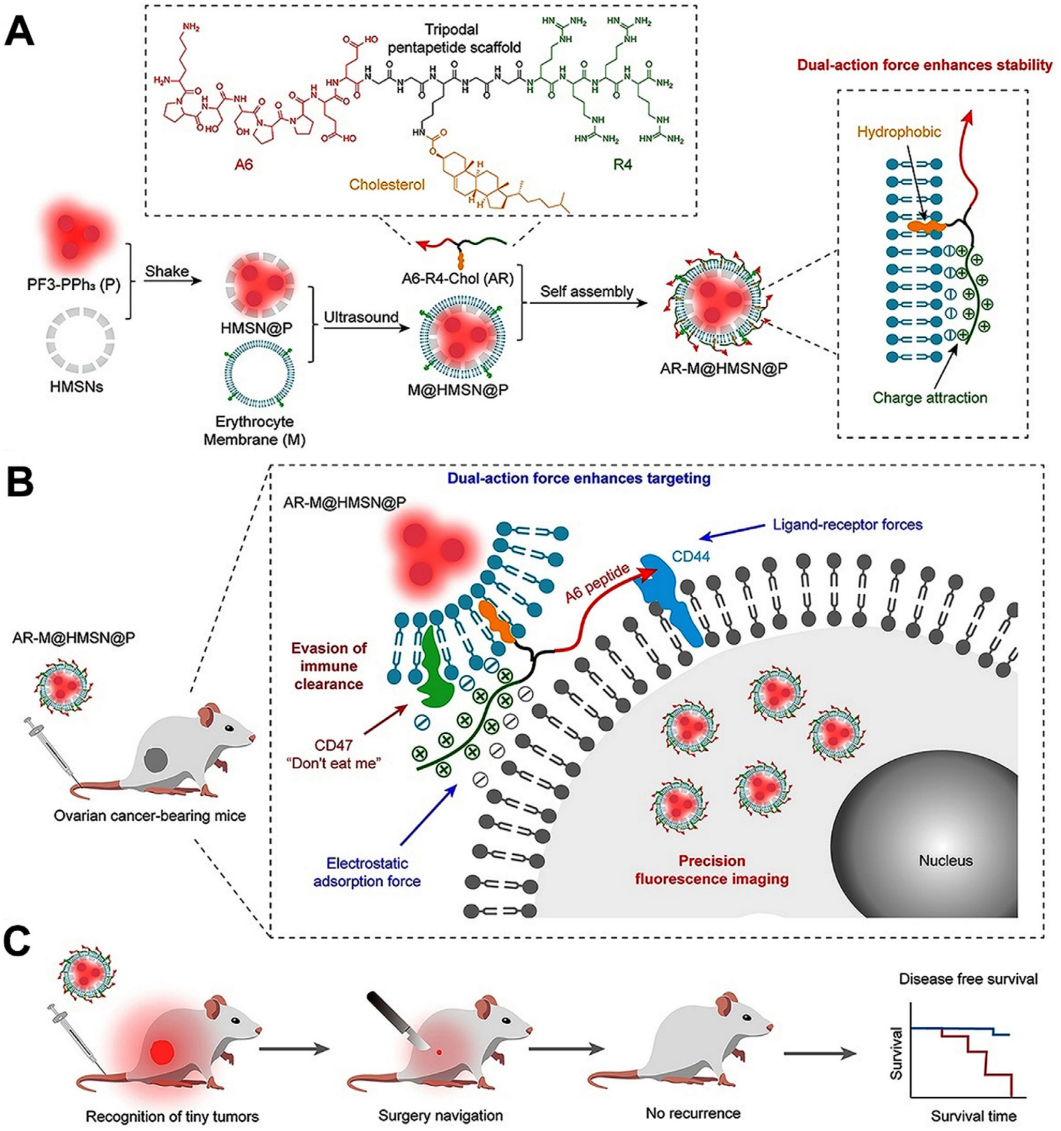
The preparation and imaging mechanisms of AR‐M@HMSN@P bioprobe. A) The preparation process of AR‐M@HMSN@P. B) The interaction mechanisms between AR‐M@HMSN@P and cancer cells in vivo. C) Fluorescence imaging‐mediated small tumor resection. Reproduced with permission.^[^ ^62^ ^]^ Copyright 2024, Springer Nature.

Tumor cell membrane proteins are the most common biomarkers for tumor theranostics. Human epidermal growth factor receptor 2 (HER2), an epithelial‐derived marker, is widely used in the diagnosis and treatment of breast cancer. The 2Rs15d nanobody obtained from dromedary inoculation of the recombinant HER2‐Fc protein targeting HER2 has been developed as a positron emission tomography (PET) diagnostic agent with good imaging properties for breast cancer.^[^ ^60^ ^]^ The CSC markers CD44 and CD133 are important targets for the detection of malignant tumors.^[^ ^61^ ^]^ As exhibited in **Figure** **4**A, Dai et al. designed a peptide‐modified fluorescent imaging bioprobe known as AR‐M@HMSN@P.^[^ ^62^ ^]^ This bioprobe uses AIEgen‐loaded hollow mesoporous silica nanoparticles as the core, and a CD44‐targeted peptide‐modified erythrocyte membrane as the shell. The “don't eat me” signal from the erythrocyte membrane, combined with CD44 targeting, efficiently promotes the entry of the nanoparticles into cells (Figure 4B). Finally, precise imaging and guidance for small tumor resection have been achieved using this system (Figure 4C).

#### 2.3.2 Immune Cell Targeting

**Figure 5:**
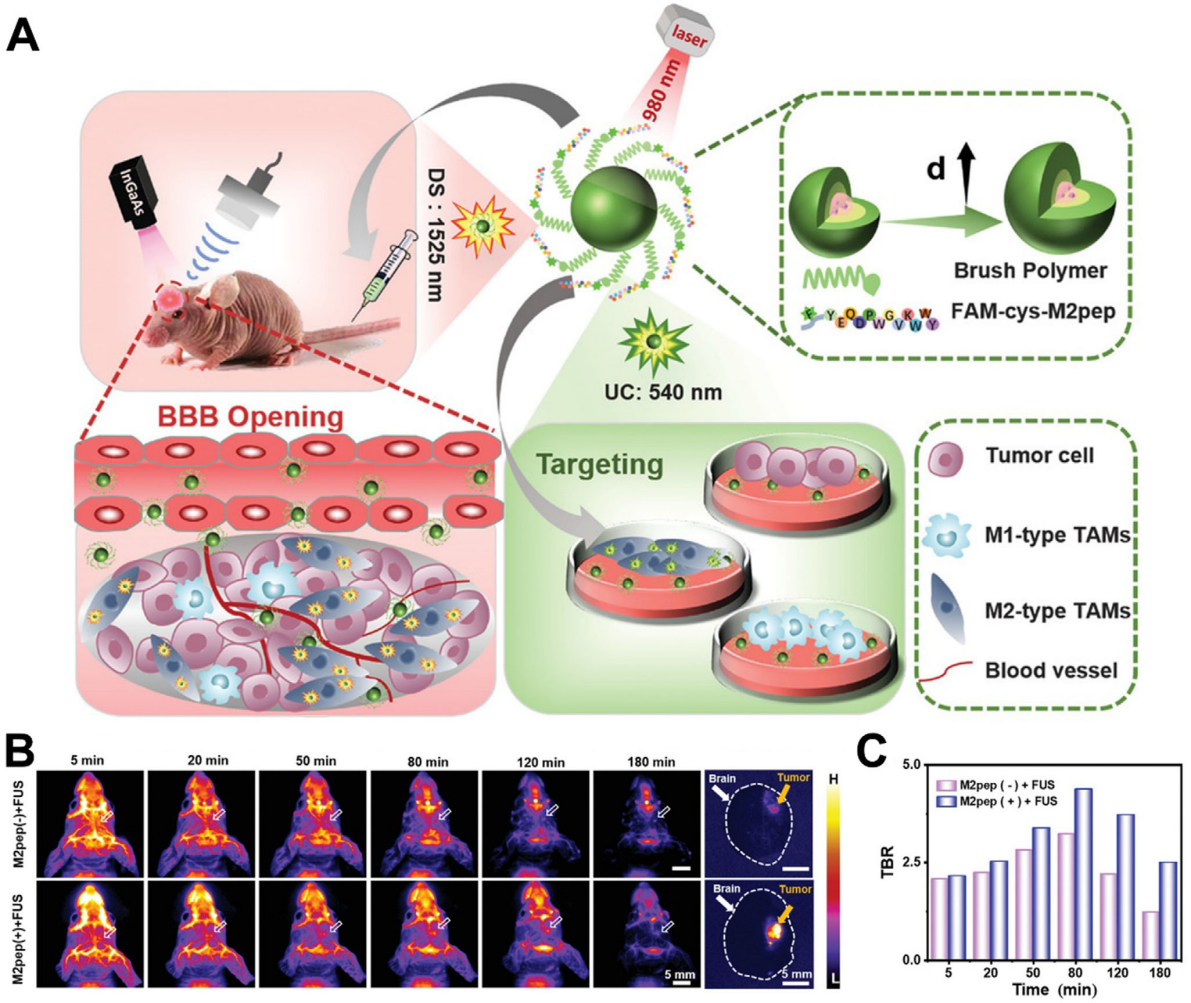
A) Mechanism diagram of the M2‐targeting nanoprobes in the orthotopic GBM mouse model. B) The near‐infrared (NIR)‐II fluorescence imaging of the nanoprobe with or without targeting ability in the orthotopic GBM‐bearing mouse model at different times. C) The TBR in (B) measured by ImageJ software. Reproduced with permission.^[^ ^63^ ^]^ Copyright 2022 Wiley‐VCH.

In addition to targeting relevant markers of tumor cells, there are many key biomarkers of immune cells. M2‐type tumor‐associated macrophages (TAMs) are closely associated with poor tumor prognosis, tumor cell invasion, and metastasis. As shown in **Figure** **5**, Li et al. designed a nanoprobe to specifically image M2‐type TAMs based on the M2‐targeting peptide YEQDPWGVKWWY (M2pep) (Figure 5A).^[^ ^63^ ^]^ Under excitation with a 980 nm laser, the nanoprobe with M2pep showed stronger fluorescence and a higher tumor‐to‐background ratio (TBR) than that of the non‐targeted group in orthotopic glioblastoma (GBM)‐bearing mouse models (Figure 5B,C), indicating that it has the ability of efficient enrichment and effective imaging at the tumor site. Similarly, Tian et al. prepared a noninvasive probe named [Ga]‐DOTA‐M2pep to achieve TAM imaging using M2pep.^[^ ^64^ ^]^ Their study revealed that radiolabeled [Ga]‐DOTA‐M2pep reached the tumor site rapidly and specifically, and also showed high levels of accumulation in B16F10 tumors at 1 h post‐injection.

#### 2.3.3 Cancer‐Associated Fibroblasts and Tumor Vascular Targeting

In addition to tumor and immune cells, cancer‐associated fibroblasts (CAFs) and the tumor vasculature are important components of tumor tissue. CAFs have been shown to play a tumor‐promoting role by promoting inflammation, inducing cancer angiogenesis and metastasis, and remodeling the extracellular matrix (ECM).^[^ ^65^ ^]^ Fibroblast activation protein (FAP), a typical CAF biomarker, has been extensively studied.^[^ ^66^ ^]^ FAP‐2286 is a DOTA‐chelator‐modified cyclic FAP‐binding peptide.^[^ ^67^ ^]^ Recently, imaging and therapeutic agents utilizing FAP‐2286‐chelated radioactive metal elements have been rapidly developed.^[^ ^67^ ^]^ Tumor angiogenesis is closely related to the escape and development of tumor cells, and this process has a multitude of potential targets.^[^ ^68^ ^]^ Integrin receptors present on the cell surface play an important role in tumor angiogenesis and metastasis and are considered to be suitable targets in biomedicine.^[^ ^69^ ^]^ The RGD (Arg‐Gly‐Asp) peptide sequence is a representative targeting peptide for integrin receptors.^[^ ^69^ ^]^ A large number of RGD peptides and their derivatives have been developed to selectively target tumor angiogenesis in the past two decades. It has been reported that the binding of RGD peptides to integrin receptors can inhibit the expression of glycoproteins in the ECM, which leads to decreased cell adhesion and tumor formation.^[^ ^70^ ^]^ Aminopeptidase N and CD13 participate in peptide chain cleavage to promote tumor vascularization. Asn‐Gly‐Arg can specifically bind to CD13 in tumor blood vessels, enabling the efficient diagnosis and treatment of tumors.^[^ ^71^ ^]^ The transferrin receptor (TfR) and low‐density lipoprotein receptor protein 1 (LRP‐1) are both highly expressed on brain capillary endothelial cells and glioma cells. TfR and LRP‐1 targeting peptide‐modified iron oxide nanoparticles can traverse the blood–brain barrier by transcytosis and target glioma cells by recognizing TfR or LRP‐1.^[^ ^72^ ^]^

Because of the overexpression of lesion‐tissue‐specific markers, targeted peptides can guide drugs to various lesion locations, thereby reinforcing the efficiency of drugs and reducing side effects.^[^ ^73^ ^]^ In addition to targeting peptides, antibodies, and aptamers also have good targeting capabilities. Compared with antibodies and aptamers, targeting peptides have the advantages of a small size, easy chemical modification, low production cost, and low immunogenicity; however, their affinity needs to be further optimized. The selection of targeting molecules in different application scenarios, including the design of radiosensitizers and radiopharmaceuticals, requires a comprehensive consideration of target characteristics, cost‐effectiveness, and other factors.

### 2.4 Self‐Assembly Performance

#### 2.4.1 Enzyme‐Induced Self‐Assembly

**Figure 6:**
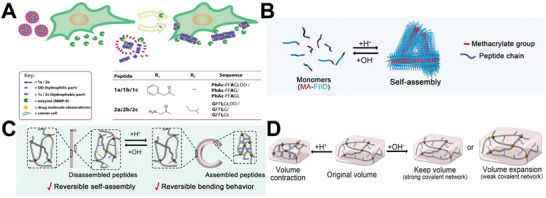
A) The micelle‐to‐fiber transition in tumor cells triggered by MMP‐9 and the alterations in the chemical structure of self‐assembled peptides in response to MMP‐9. Reproduced with permission.^[^ ^77^ ^]^ Copyright 2016, Elsevier Ltd. B) The schematic of MA‐FIID monomer and self‐assembly. C) Reversible deformation of pH‐responsive self‐assembly in response to pH change. D) The expansion and shrinkage of hydrogel under different pH conditions. Reproduced with permission.^[^ ^80^ ^]^ Copyright 2023, Wiley‐VCH.

As endogenous stimuli, enzymes work directly on the chemical bonds between peptides or between peptides and bridging molecules. Under enzymatic stimulation, peptide‐based biomaterials can be modified to fulfill their required functions. There are a large number of enzymes in the human body, including matrix metalloproteinases (MMPs),^[^ ^74^ ^]^ alkaline phosphatases (ALPs), carboxylesterases, enterokinases, cathepsin B, gelatinases, and caspases.^[^ ^75^ ^]^ MMPs have been identified as key molecular targets in various cancers and participate extensively in many reactions.^[^ ^76^ ^]^ As depicted in **Figure** **6**A, Ulijn et al. designed a self‐assembled peptide‐based nanostructure responsive to matrix metalloproteinases (MMP‐9). It can transform from micelles to nanofibers and transport the encapsulated doxorubicin to tumor sites with MMP‐9 overexpression.^[^ ^77^ ^]^ Importantly, the formation of the nanostructure effectively improved the tumor inhibition efficacy of doxorubicin. In another experiment, a self‐assembled peptide containing the peptide sequences PLGLAG and RGD was designed, which formed a spherical structure carrying drugs to prolong the retention of drugs at the tumor site via the EPR effect and transformed into aggregates under the catalysis of MMP‐2 to release drugs.^[^ ^78^ ^]^ Moreover, Bai et al. reported an amphiphilic peptide, Ac‐VVVVVVKKK‐NH_2_ (V_6_K_3_), which transformed from nanoparticles to nanofibers via plasma amine oxidase, thereby effectively achieving the loading and release of drugs.^[^ ^79^ ^]^

#### 2.4.2 pH‐Induced Self‐Assembly

The pH of most regions within the human body is approximately neutral; however, some lesion tissues have acidic pH, which also provides opportunities for the self‐assembly of pH‐responsive peptides. Basic amino acids, including histidine (H), lysine (K), and arginine (R), carry a positive charge at pH levels below the p*K*_a_ of their respective side chains, whereas acidic amino acids, including glutamic acid (E) and aspartic acid (D), carry a negative charge at pH levels above the p*K*_a_ of their side‐chain carboxylates. H, K, R, E, and D are ionizable amino acids commonly used when designing pH‐responsive peptides, as they can undergo protonation or deprotonation in acidic environments, leading to alterations in the structure of the assembly.^[^ ^81^ ^]^ For example, an ultra‐pH‐responsive peptide nanocarrier comprising a pH‐sensitive block with three H residues was reported to trigger stepwise dissociation of nanoparticles in an acidic microenvironment.^[^ ^82^ ^]^ Furthermore, a smart hydrogel was constructed using pH‐sensitive self‐assembled peptides (MA‐FIID) and poly (N‐isopropylacrylamide) [PNIPAM] backbones.^[^ ^80^ ^]^ MA‐FIID has a flexible response to different pH environments, driving the disassembly and assembly of the peptides, which is reflected in the expansion and shrinkage of the hydrogel (Figure 6B,C). Interestingly, the hydrogels showed good shape memory, including temporary shape retention, shape recovery, and complex deformation of the PNIPAM skeleton caused by salt and pH stimulation (Figure 6D). This strategy offers new insights into the construction of intelligent soft materials based on peptides with stimulus responses.

#### 2.4.3 Redox‐Reaction‐Induced Self‐Assembly

**Figure 7:**
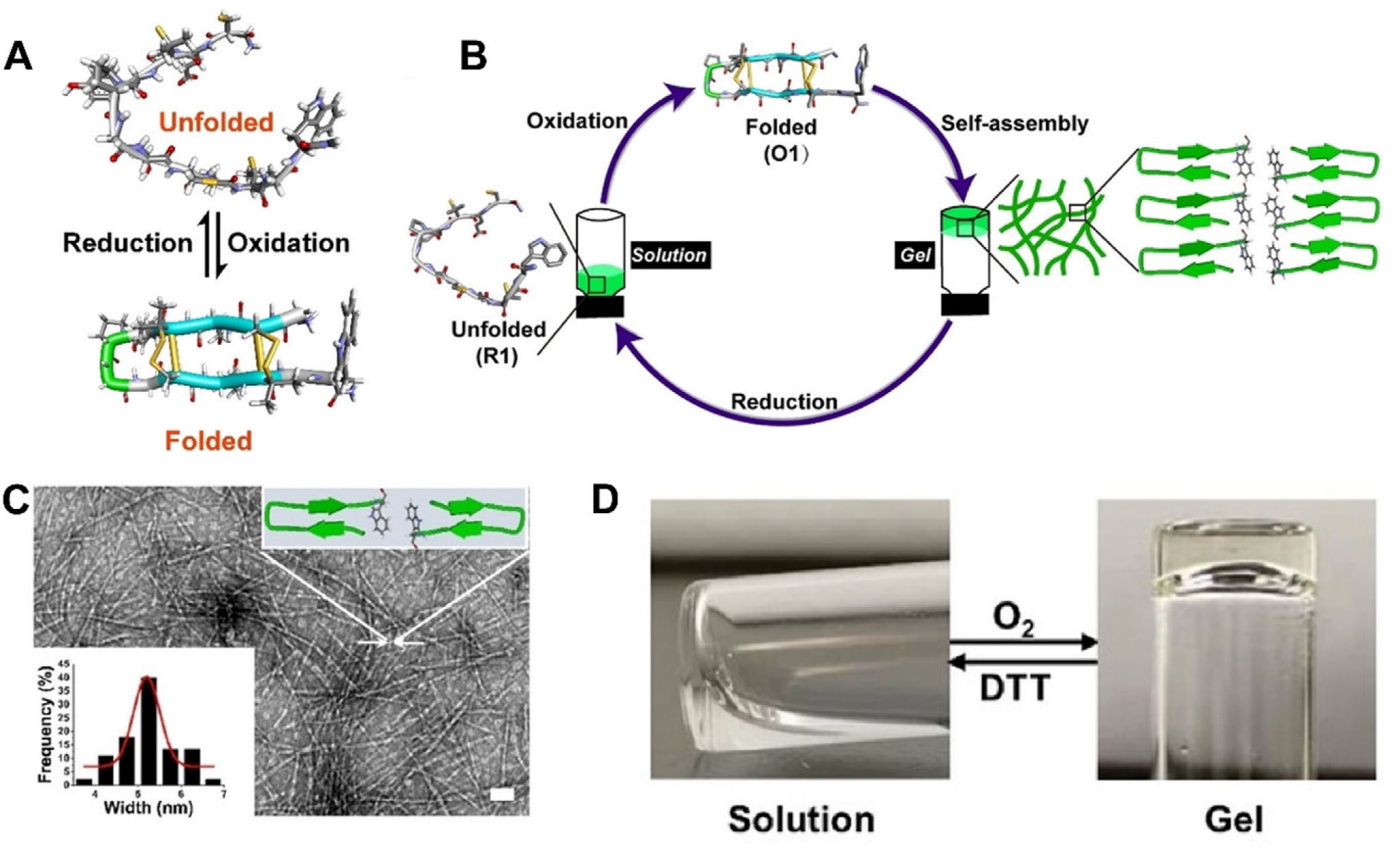
A) The conformation transition of the disulfide‐rich peptide between reduction and oxidation. B) Schematic depiction of cyclic changes in solution and gel states of this peptide. C) TEM and dynamic light scanning images of the gel. D) Optical photographs of the reversible Gel‐to‐Sol transition. Reproduced with permission.^[^ ^87^ ^]^ Copyright 2022, Wiley‐VCH.

The TME is characterized by a heterogeneous redox state owing to the aberrant production of glutathione (GSH) and reactive oxygen species (ROS).^[^ ^83^ ^]^ The types of tumors and even different stages and regions of the same tumor can have different levels of ROS and GSH.^[^ ^84^ ^]^ Niu et al. reported supramolecular aggregates consisting of cucurbituril, methionine‐containing amphiphilic peptides, and perylene diimide. When ROS oxidizes methionine residues in an amphiphilic peptide to sulfoxides or sulfones, the binding affinity within the supramolecular aggregates is influenced, thereby changing them from nano‐sized particles to micron‐sized sheets.^[^ ^85^ ^]^ Mo et al. reported redox‐regulated in situ seed‐induced co‐assembly between a redox‐resistant seed and a redox‐responsive assembly monomer. Of note, in cancer cells with high levels of GSH, the seed can accelerate the co‐assembly, offering a new method for developing biomedical materials in living systems.^[^ ^86^ ^]^ As shown in **Figure** **7**, Shi et al. designed a disulfide‐rich peptide containing two cysteine and penicillamine residues (Figure 7).^[^ ^87^ ^]^ This peptide underwent conformational changes under oxidative and reducing conditions (Figure 7A). The oxidized form of the peptide has a regular β‐hairpin‐like structure, and the reduced form of the peptide has a random structure, which leads to the peptides exhibiting cyclic changes in solution and gel states (Figure 7B). Evident fibrils of the assembling peptide after oxidation were observed by transmission electron microscopy (Figure 7C). Of note, the morphological transition of the sol–gel occurs when exposed to air, whereas a reversible change occurs under dithiothreitol treatment (Figure 7D). This strategy potentially enables on‐demand drug release.

#### 2.4.4 Self‐Assembly Induced by Other Factors

Among many exogenous stimuli, photo‐stimulation offers the advantages of non‐invasiveness and high spatiotemporal selectivity. Zhang et al. constructed a peptide‐antibiotic conjugate to generate nanoantibacterial on bacterial surfaces triggered by NIR light.^[^ ^88^ ^]^ Wu et al. developed smart peptide‐assembled nanomaterials with nanosphere–nanofiber–nanosphere transitions that are dually responsive to the acidic TME and near infrared laser irradiation.^[^ ^89^ ^]^ In addition, temperature has been widely studied as an influencing factor in peptides that undergo structural changes and self‐assembly. ELPs designed by Barker et al. can respond to temperature stimuli by transitioning from micelles to fibrinogen and finally to microscale particles with temperature changes.^[^ ^90^ ^]^

**Figure 8:**
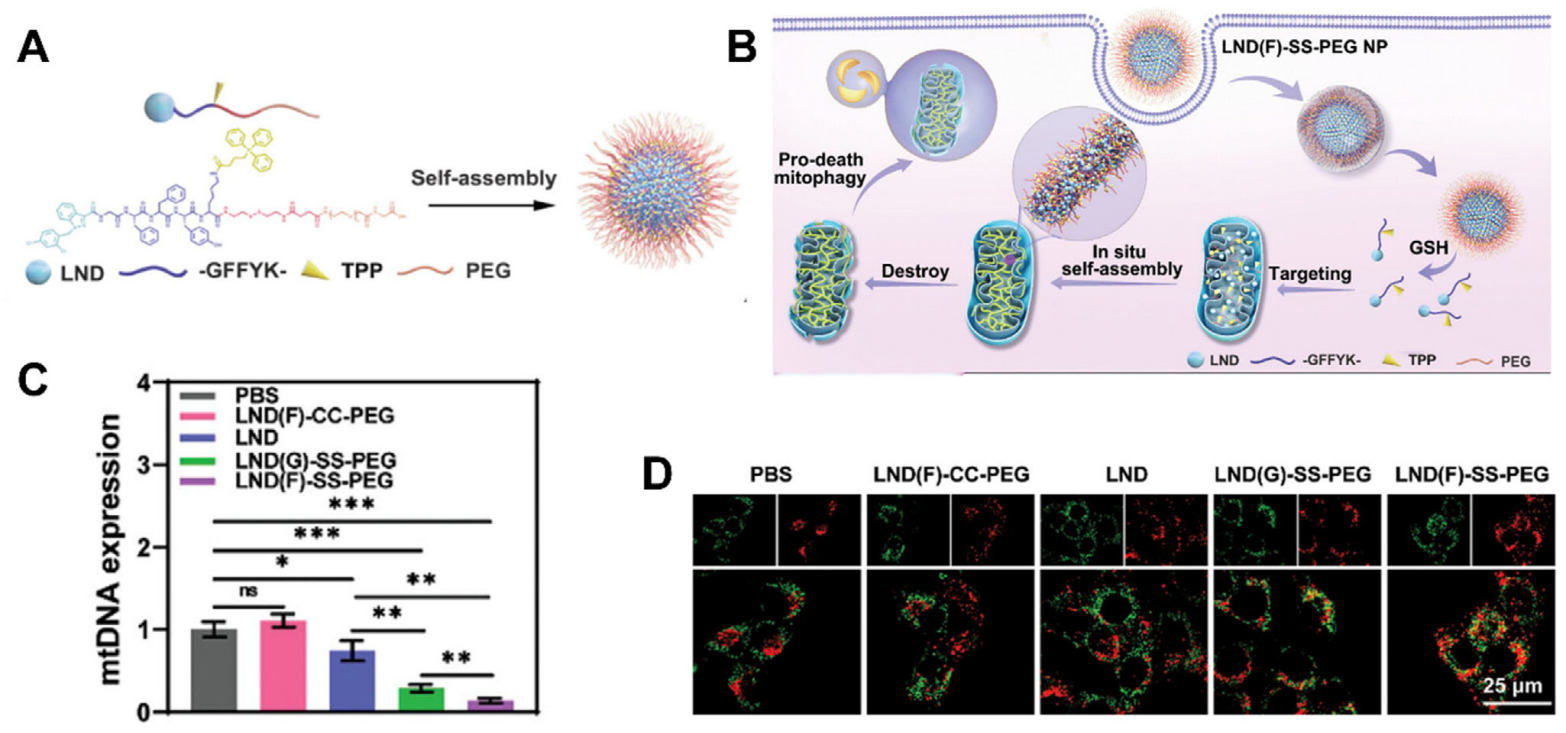
A) Chemical structure of LND(F)‐SS‐PEG. B) Scheme illustration of the cascade‐responsive process on mitochondria of the peptide‐based drug. C) qRT‐PCR analysis of mitochondrial DNA expression. D) Co‐localization images of lysosomes (red) with mitochondria (green). Antitumor efficacy with RT of LND(F)‐SS‐PEG enhances radiotherapy. Reproduced with permission.^[^ ^92^ ^]^ Copyright 2024, Wiley‐VCH.

In addition to the aforementioned pH, enzymes, ROS, temperature, and light stimuli that can modulate the behavior of peptides, some biological structures are also important for stimulating peptide assembly.^[^ ^91^ ^]^ As illustrated in **Figure** **8**, Liu et al. reported a mitochondria‐localized in situ self‐assembly peptide‐based drug delivery system.^[^ ^92^ ^]^ The drug precursor, LND(F)‐SS‐PEG, consists of a mitochondria‐targeting peptide, polyethylene glycol, and the model drug lonidamine, which can self‐assemble into nanomicelles in vitro (Figure 8A). After being endocytosed into the cell, the micelles undergo a cascade‐responsive process, including GSH‐responsive drug release, mitochondria‐targeted enrichment, and in situ self‐assembly in the mitochondria (Figure 8B). The organelle‐specific self‐assembly system effectively destroys mitochondria (Figure 8C), promotes pro‐death mitophagy (Figure 8D), improves the immunosuppressive TME, and enhances tumor sensitivity to RT. The group also synthesized and developed a bacterial‐surface‐induced self‐assembling peptide derivative, I‐Rho‐FF‐Van, which effectively detects and images bacterial infections in situ.^[^ ^93^ ^]^

Peptide self‐assembly is a spontaneous process primarily driven by thermodynamics and kinetics. The nanostructure and stabilizing arrangements in the self‐assembled systems, such as nanoparticles, nanofibers, or other structures with regular morphology and uniform size, are formed through intermolecular non‐covalent interactions, including π–π stacking, charge interactions, van der Waals interactions, hydrophobic interactions, and hydrogen bonding.^[^ ^18^ ^]^ By reasonably designing the molecular sequence of peptides, peptide assemblies can form well‐defined architectures. The adaptive self‐assembly properties of peptide‐based biomaterials are expected to meet the requirements of personalized and precise RT.

## 3 Peptide‐Based Radiosensitizers

RT is extensively used as a therapeutic approach in clinical cancer therapies. However, the long‐term application of RT is limited by shortcomings related to biological alterations in the tumor and the surrounding microenvironment. The main factors affecting RT include an altered cell cycle, repopulation by CSCs, hypoxia, altered management of oxidative stress, evasion of apoptosis, an altered DNA damage response, enhanced DNA repair, inflammation, and abnormal energy mechanisms. Given these complex RT resistance mechanisms, radiosensitizers are urgently needed to improve the treatment response and patient prognosis.^[^ ^95^ ^]^ Peptide‐based biomaterials have the advantages of peptides themselves, including good biocompatibility, ease of synthesis and modification, and the nanoparticles formed also have the advantages of multiple drug loading and EPR effects. Peptide‐based biomaterials have been studied and applied as direct radiosensitizers or carriers for radiosensitizers to enhance radiotherapeutic efficacy. In the next section, the application of peptide‐based biomaterials to enhance RT is described.

### 3.1 Peptides as Direct Radiosensitizers

#### 3.1.1 DNA Repair Inhibition

**Figure 9:**
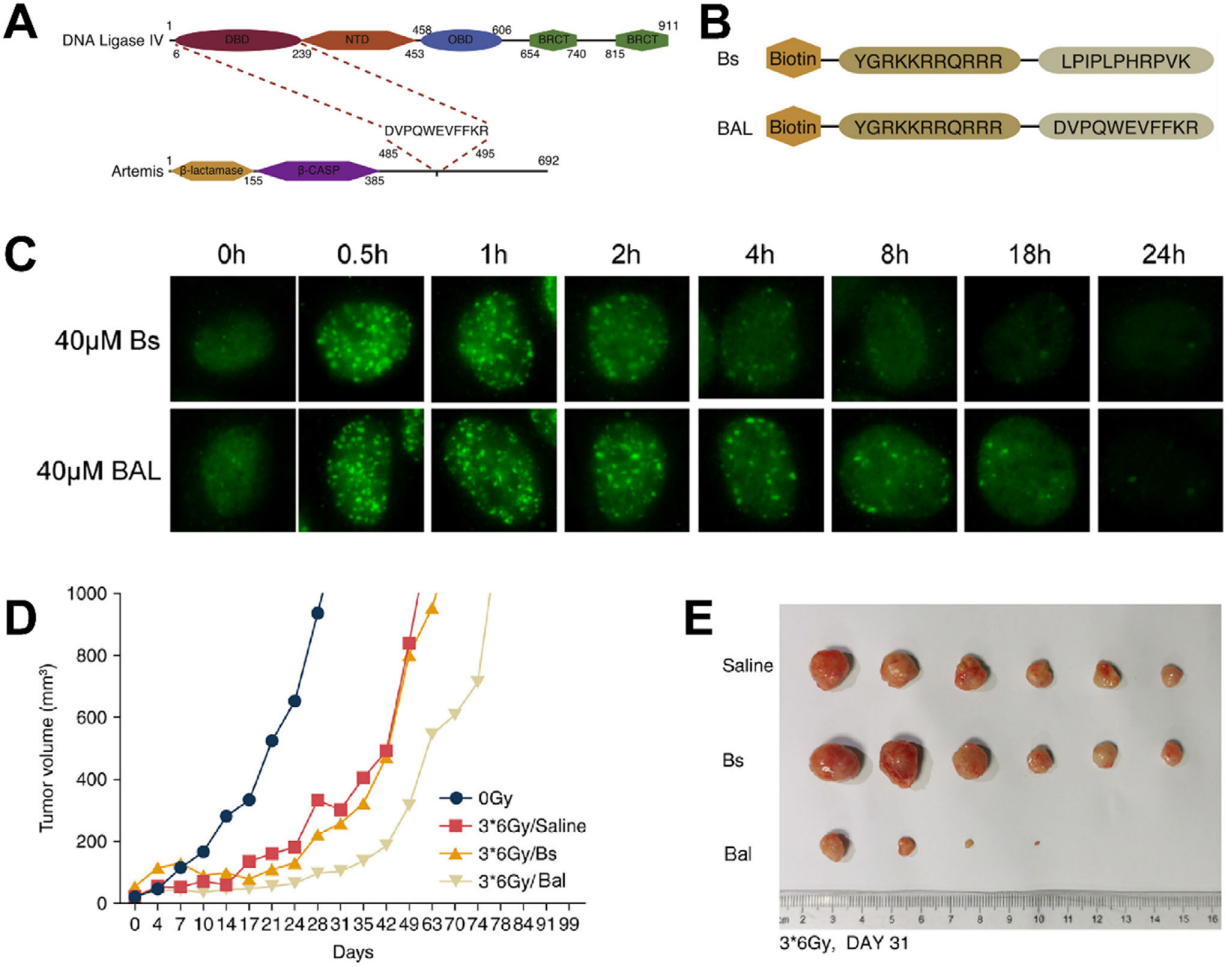
A) Schematic representation of the interacting regions of Artemis and DNA Ligase IV. B) Amino‐acid composition of BAL and its control group. C) Immunofluorescent γ‐H2AX staining of Hela cells treated with different interventions with 4 Gy radiation. D) Tumor volume changes after various treatments. E) Photographs of excised tumors after 31 days of treatment. Reproduced with permission.^[^ ^97^ ^]^ Copyright 2021, Elsevier Inc.

Since DNA is the main target of ionization radiation (IR)‐induced cell death,^[^ ^96^ ^]^ targeting the DNA damage response pathways that affect the radiosensitivity of tumor cells is a promising approach. Artemis and DNA Ligase IV are two critical elements in DNA repair, and inhibiting either of them may achieve radiosensitization. According to the fact that Artemis interacts with DNA Ligase IV to induce DNA repair via its crystal structure of DVPQWEVFFKR (**Figure** **9**A), Sun et al. designed and synthesized an inhibitory peptide BAL containing the sequence of DVPQWEVFFKR to block the interaction between DNA Ligase IV and Artemis and inhibit the DNA repair (Figure 9B).^[^ ^97^ ^]^ Compared with the control group without DVPQWEVFFKR, BAL exhibited prolonged DNA repair time based on the results of γ‐H2AX staining (Figure 9C). BAL and irradiation synergistically inhibited tumor growth in xenograft mouse models (Figure 9D and E). Zheng et al. found that biotin‐labeled peptide 3 (BTW3) targets the autophosphorylation of DNA‐PKcs at threonine 2647. Considering that DNA‐PKcs threonine 2647 is a key element participating in the DNA damage repair process in response to IR, BTW3 may have the potential to be developed as a radiosensitizer.^[^ ^98^ ^]^

#### 3.1.2 Immune and Senescence‐Like Cell Targeting

RT and immunity are closely linked, and RT can produce strong immune effects. Programmed cell death 1 (PD‐1) signaling and its ligand PD‐L1 are classical immune checkpoints, and related inhibitors have achieved immense success in clinical trials.^[^ ^99^ ^]^ A clinical trial reported that PD‐1/PD‐L1 inhibitors could be potential radiosensitizers in head and neck squamous cell carcinoma,^[^ ^100^ ^]^ and Liu et al. synthesized a P‐peptide using Fmoc‐protected d‐amino acids to target and disrupt the function of PD‐L1 in tumor cells.^[^ ^101^ ^]^ In addition to PD‐1 and PD‐L1, the immune checkpoint inhibitor targeting CTLA‐4 also exhibits some radiosensitization effects in patients with melanoma and non‐small‐cell lung cancer.^[^ ^102^ ^]^ A synthetic peptide that binds to the CTLA‐4 protein to block the contact of the CTLA‐4 receptor with B7‐1 has also been reported.^[^ ^103^ ^]^

**Figure 10:**
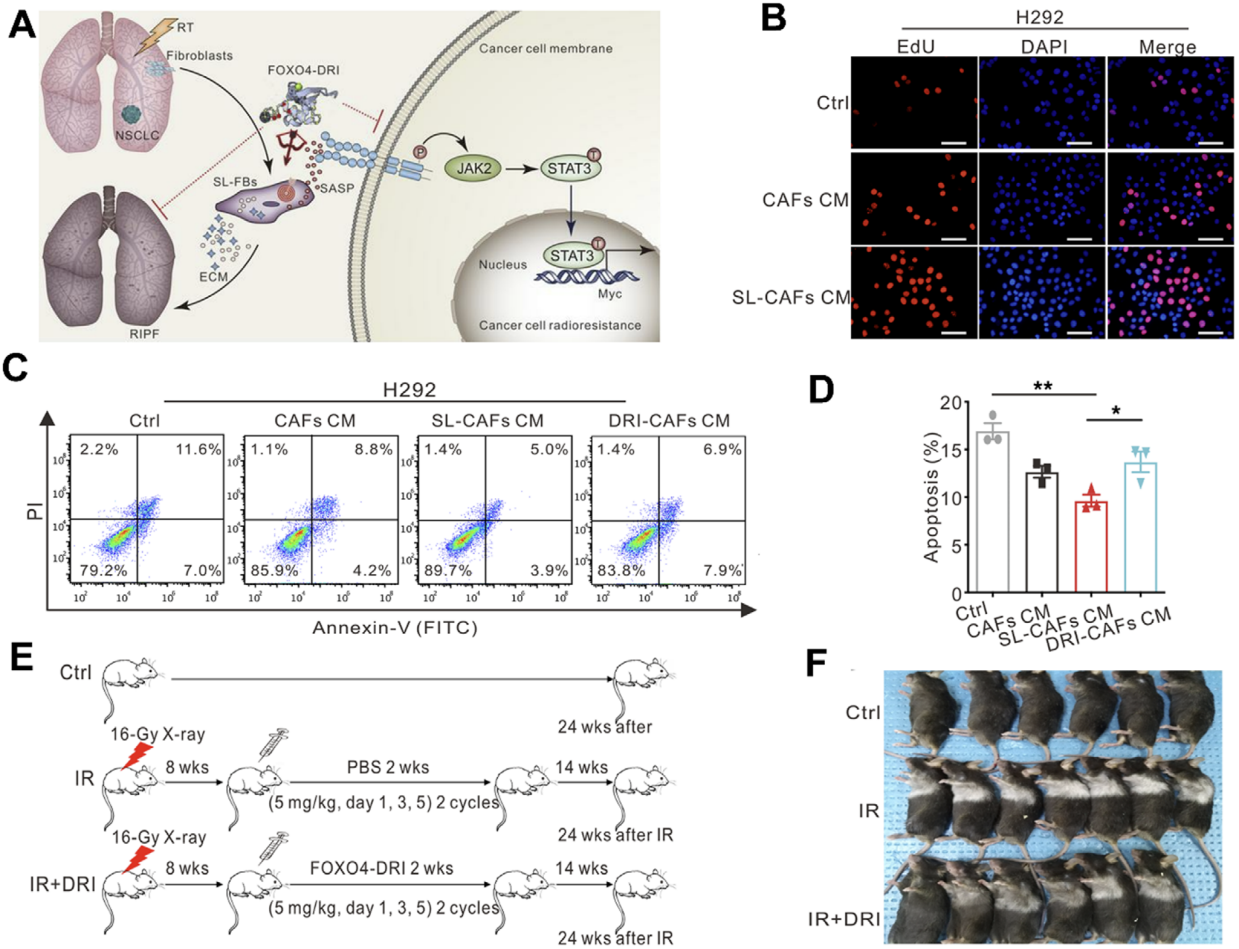
A) Therapeutic mechanisms of FOXO4‐DRI specifically induce senescence‐like CAFs apoptosis. B) Fluorescence images of EdU staining in H292 cells cultured with the blank medium (control), CAFs CM, and SL‐CAFs CM. C) Flow cytometry images of cell apoptosis and D) its quantitative data of the apoptosis rates after different treatments plus 8 Gy irradiation. E) The schedule of animal therapy for radiation‐induced pulmonary fibrosis (RIPF). F) Photographs of the mice with RIPF after treatment with different formulations. Reproduced with permission.^[^ ^104^ ^]^ Copyright 2021, The American Society for Clinical Investigation.

In addition to immune cells, radiation‐induced senescence‐like CAFs are another target for radiosensitization. Yang et al. identified that senescence‐like CAFs could promote radioresistance through activating the JAK/STAT pathway. Thus, they uncovered a FOXO4‐p53‐interfering peptide (FOXO4‐DRI) to induce the apoptosis of senescence‐like CAFs and achieve radiosensitization (**Figure** **10**A).^[^ ^104^ ^]^ In cell proliferation assays, cancer cells cultured with conditioned media of senescence‐like CAFs (SL‐CAFs CM) show higher EdU fluorescence than those cultured with normal CAF CM, indicating stronger proliferative ability (Figure 10B). The FOXO4‐DRI plus IR can induce most cancer cells' apoptosis measured by flow cytometry, which exhibits a good ability of radiosensitization (Figure 10C,D). In addition to its radiosensitizing ability, FOXO4‐DRI therapy alleviates radiation‐induced pulmonary fibrosis in mouse models compared to IR alone (Figure 10E,F).

#### 3.1.3 Tumor Revascularization

Because of tumor overgrowth, the metabolic demands of the tumor far exceed the blood supply, resulting in vascular dysfunction and hypoxia in tumor cells. Thus, targeting tumor vessels to improve the hypoxic state may be a favorable strategy for decreasing radioresistance. It is reported that peptides targeting the vasculature can restore normal vessels and increase oxygen levels in TME for radiosensitization applications. As a typical example, Fuchs et al. proposed a method to improve tumor therapy by normalizing blood vessels using a C‐type natriuretic peptide derivative (dCNP).^[^ ^105^ ^]^ dCNP is a vasculature‐normalizing agonist that remodels tumor blood vessels and alleviates intertumoral hypoxia. In several mouse models of orthotopic and subcutaneous solid tumors, including colon and pancreatic adenocarcinomas, this well‐tolerated formulation stimulates the formation of highly functional tumor blood vessels, thereby reducing hypoxia. Of note, treatment with dCNP also ameliorated antitumor immune responses. The addition of dCNP to the radiation therapy regimen improves its efficacy against solid tumors in immunocompetent mice. In addition, Liu et al. claimed that the heme‐targeting drugs, heme‐sequestering peptide 2 (HSP2) and cyclopamine tartrate (CycT), could normalize the tumor vasculature and improve tumor oxygenation.^[^ ^106^ ^]^ Heme is essential for ATP generation via oxidative phosphorylation, while HSP2 inhibits heme uptake, and CycT inhibits heme synthesis. HSP2 and CycT elicit multiple tumor‐suppressing functions, including inhibiting angiogenic function, normalizing the tumor vasculature, alleviating tumor hypoxia, and inhibiting oxygen consumption and ATP generation.

### 3.2 Peptide‐Based Biomaterials as the Carriers of Radiosensitizers

The therapeutic benefits of RT in many tumor treatments remain unsatisfactory and are challenged by resistance to RT. Some radiosensitizers to overcome radioresistance are usually limited by the complex tumor microenvironment and cannot achieve effective delivery. Thus, delivery carriers are necessary to distribute radiosensitizers in tumors. Recently, self‐assembled peptide‐based drug delivery systems have emerged for radiosensitization, providing new opportunities for the development of radiosensitizers.^[^ ^107^ ^]^

#### 3.2.1 Enzyme‐Induced Peptide Carriers

Enzyme‐induced self‐assembly is crucial for the development and design of peptide‐based carriers. For example, a micelle encapsulating bFGF (bFGF@K2) and containing a precursor peptide, K2, has been designed.^[^ ^108^ ^]^ Upon cleavage of MMP‐9, the micelles are reconstructed into nanofibers, and bFGF is delivered into tumors with a longer retention time. In addition, Liang et al. constructed a porphyrin‐conjugating smart peptide that self‐assembled into porphyrin nanofibers after cleavage by caspase‐3. The formation of porphyrin‐loaded nanofibers magnified apoptosis and antitumor effects.^[^ ^109^ ^]^

**Figure 11:**
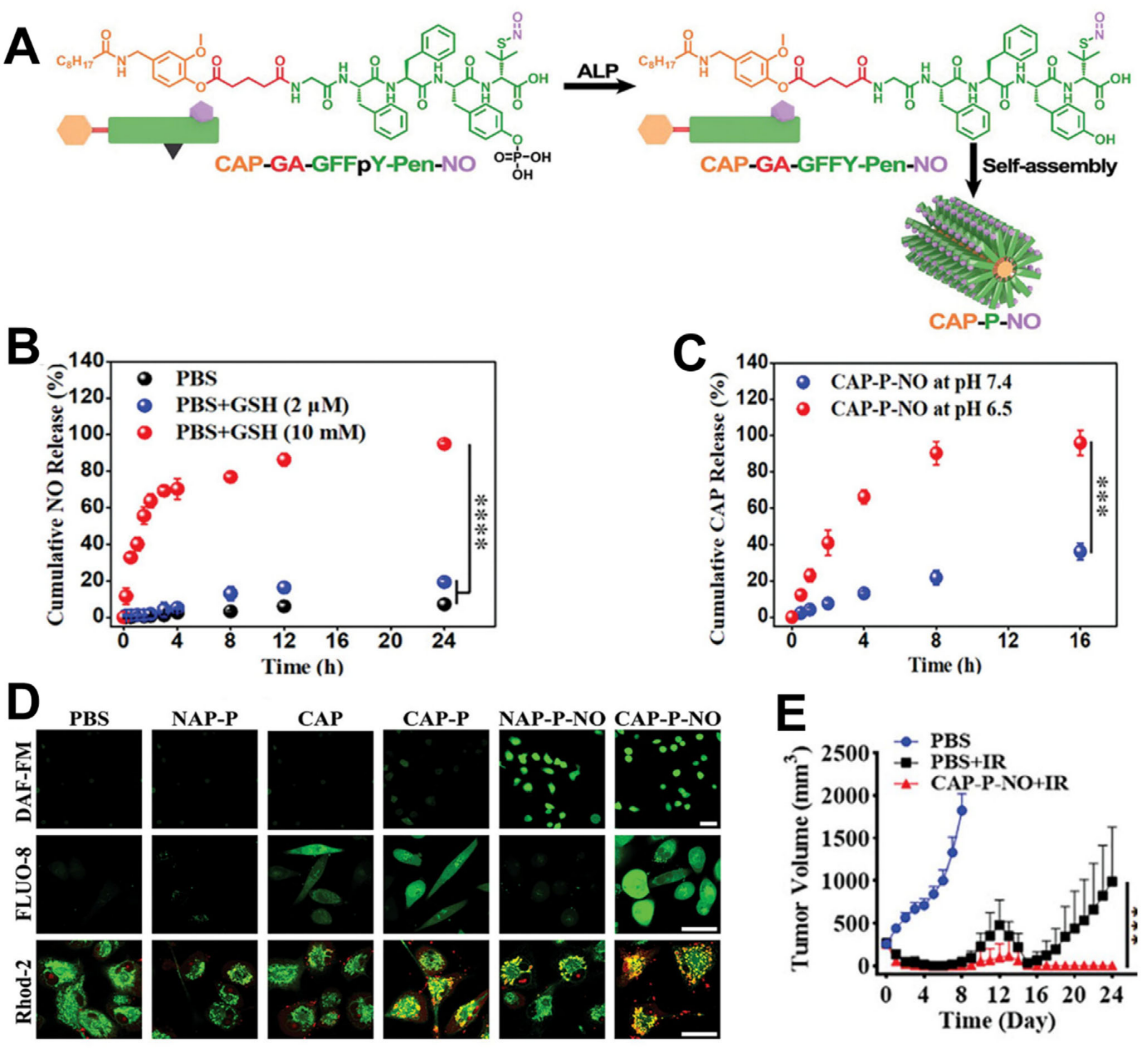
A) The preparation of CAP‐P‐NO nanofibers. B) The cumulative NO release from CAP‐P‐NO with or without GSH treatment. C) The cumulative CAP release from CAP‐P‐NO under different pH conditions. D) CLSM images of the Ca^2+^ (red) and mitochondria (green). E) Tumor volumes after indicated treatments in the mice bearing a patient‐derived hepatic tumor. Reproduced with permission.^[^ ^111^ ^]^ Copyright 2024, Wiley‐VCH.

Notably, ALP plays a significant role in guiding the self‐assembled peptide system, especially in improving the targeting function, achieving morphological transformation, and promoting radiosensitization.^[^ ^110^ ^]^ As depicted in **Figure** **11**A, our group developed a cananomodulator (CAP‐P‐NO) consisting of a self‐assembled peptide integrating capsaicin (CAP) and nitric oxide (NO), which self‐assembled into dense nanofiber structures under ALP catalysis.^[^ ^111^ ^]^ When CAP‐P‐NO reached the tumor site, CAP and NO were released in response to intracellular GSH and the extracellular pH (Figure 11B,C). Intracellular calcium homeostasis is closely associated with the aberrant proliferation, metastatic dissemination, and drug resistance of tumor cells.^[^ ^112^ ^]^ Released CAP and NO can disrupt cellular Ca homeostasis by triggering the influx of extracellular Ca and the release of Ca from the tumor endoplasmic reticulum, thereby achieving radiosensitization and leading to the irreversible disruption of intracellular homeostasis (Figure 11D). CAP‐P‐NO potentiated the efficacy of RT in patient‐derived hepatic tumor models, with the combination treatment exhibiting the slowest growth in tumor volume of the treatments tested (Figure 11E).

Moreover, our group developed a tumor radiosensitizer, Nap‐GFFpYSV, by conjugating YSV with self‐assembling peptides.^[^ ^113^ ^]^ Nap‐GFFpYSV self‐assembles into nanofibers under the catalysis of ALP, which sensitizes cancer cells with high levels of ALP expression to radiation. ALP‐induced in situ peptide‐based nanovaccines can capture and encapsulate autologous antigens produced by radiation and exhibit effective radioimmunotherapy efficacy.^[^ ^114^ ^]^

#### 3.2.2 Physical‐Stimulation‐Induced Peptide Carriers

Compared to enzymatic reactions, physical methods are relatively easy to implement. Chen et al. reported an X‐ray‐responsive polypeptide nanogel that could achieve on‐demand delivery of chemotherapeutic agents triggered by IR, exhibiting improved RT efficacy and reduced side effects.^[^ ^115^ ^]^ Gold (Au), platinum (Pt), and other high‐Z metals have ideal radiosensitive effects because of their ability to increase intracellular radiation energy deposition.^[^ ^95^ ^]^ Coulter et al. co‐assembled a short amphipathic peptide, RALA, and gold nanoparticles (AuNPs) via electrostatic interactions.^[^ ^116^ ^]^ The presence of RALA boosted the uptake efficiency of negatively charged AuNPs by tumor cells, and co‐assembly significantly strengthened the radiotherapeutic sensitivity of prostate cancer cells compared with AuNPs alone. Moreover, we constructed a peptide‐based delivery system, N–P by co‐assembling positively charged cisplatin with negatively charged peptides.^[^ ^117^ ^]^ The formed hydrogel increased the number of Pt–DNA adducts, blocked the cell cycle, inhibited cyclooxygenase‐2, and ultimately increased the efficacy of RT.

**Figure 12:**
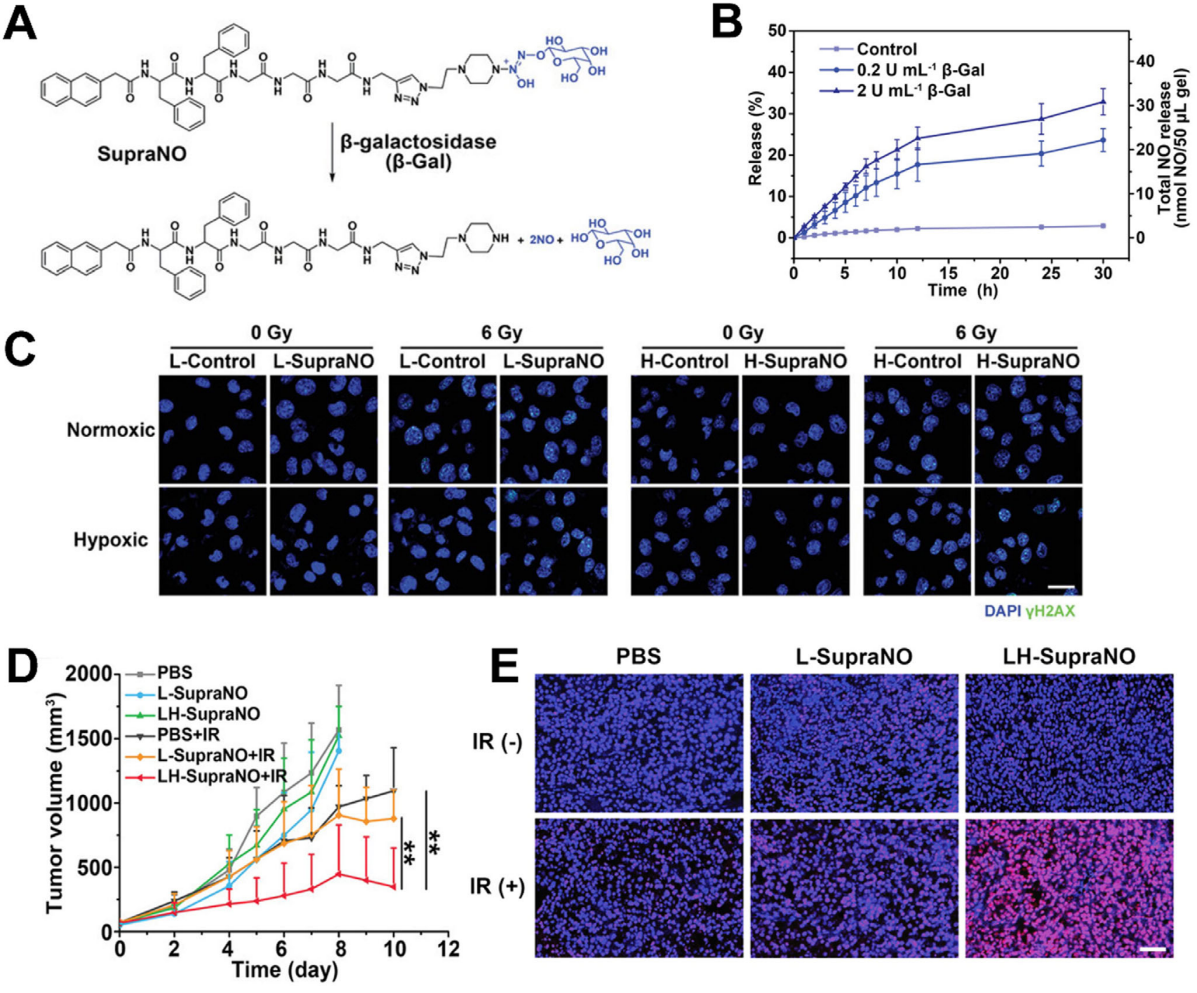
A) Chemical structure of SupraNO and the catalyzed reaction by β‐Gal. B) NO release profile from SupraNO after treatment with various concentrations of β‐Gal. C) γ‐H2AX immunofluorescence images in the tumor cell in hypoxic and normoxic conditions. D) Tumor growth curves after different treatments. E) TUNEL staining in tumor tissue sections after different treatments. Reproduced with permission.^[^ ^120^ ^]^ Copyright 2022, Wiley‐VCH.

Targeting energy metabolism pathways has been extensively exploited to enhance radiosensitivity. A temperature‐triggered mitochondria‐targeted peptide‐based nanoplatform has also been reported.^[^ ^118^ ^]^ Because of the hydrophobic double‐membrane structure and negative potential of mitochondria, this drug can target mitochondria through its cationic charge and lipophilicity. The drug enters the mitochondria to selectively attack cancer cells, resulting in a higher accumulation in cancer cells than in normal cells. Finally, this peptide‐based drug effectively inhibits the mitochondrial energy pathway, selectively killing cancer cells, and shows a prominent radiosensitization effect with a high sensitization enhancement ratio of 2.58. NO dilates blood vessels to act as an effective radiosensitizer, and many NO donors have been developed to deliver NO and render tumors more susceptible to radiation.^[^ ^119^ ^]^ Liu et al. constructed a peptide‐based supramolecular hydrogel as an NO depot (SupraNO) by heating and cooling to improve the efficacy of RT.^[^ ^120^ ^]^ This hydrogel precursor was formed by conjugating a short self‐assembling peptide with an NO prodrug (**Figure** **12**A). After incubating SupraNO with different concentrations of β‐galactosidase (β‐Gal), NO was released in a controllable manner (Figure 12B). Similarly, after incubating with SupraNO, B16 cells with high expression levels of β‐Gal produce more NO, leading to more DNA double‐strand breaks after irradiation (Figure 12C). After the hydrogel is injected into the tumor, β‐Gal in the tumor environment continuously induces the release of NO, resulting in local sustained NO delivery at a low dose, while intravenous β‐Gal can trigger a large amount of NO release. The slow and rapid release of NO is coordinated to achieve obvious tumor inhibition and RT sensitization of hypoxic tumors (Figure 12D,E).

#### 3.2.3 Redox‐Induced Peptide Carriers

**Figure 13:**
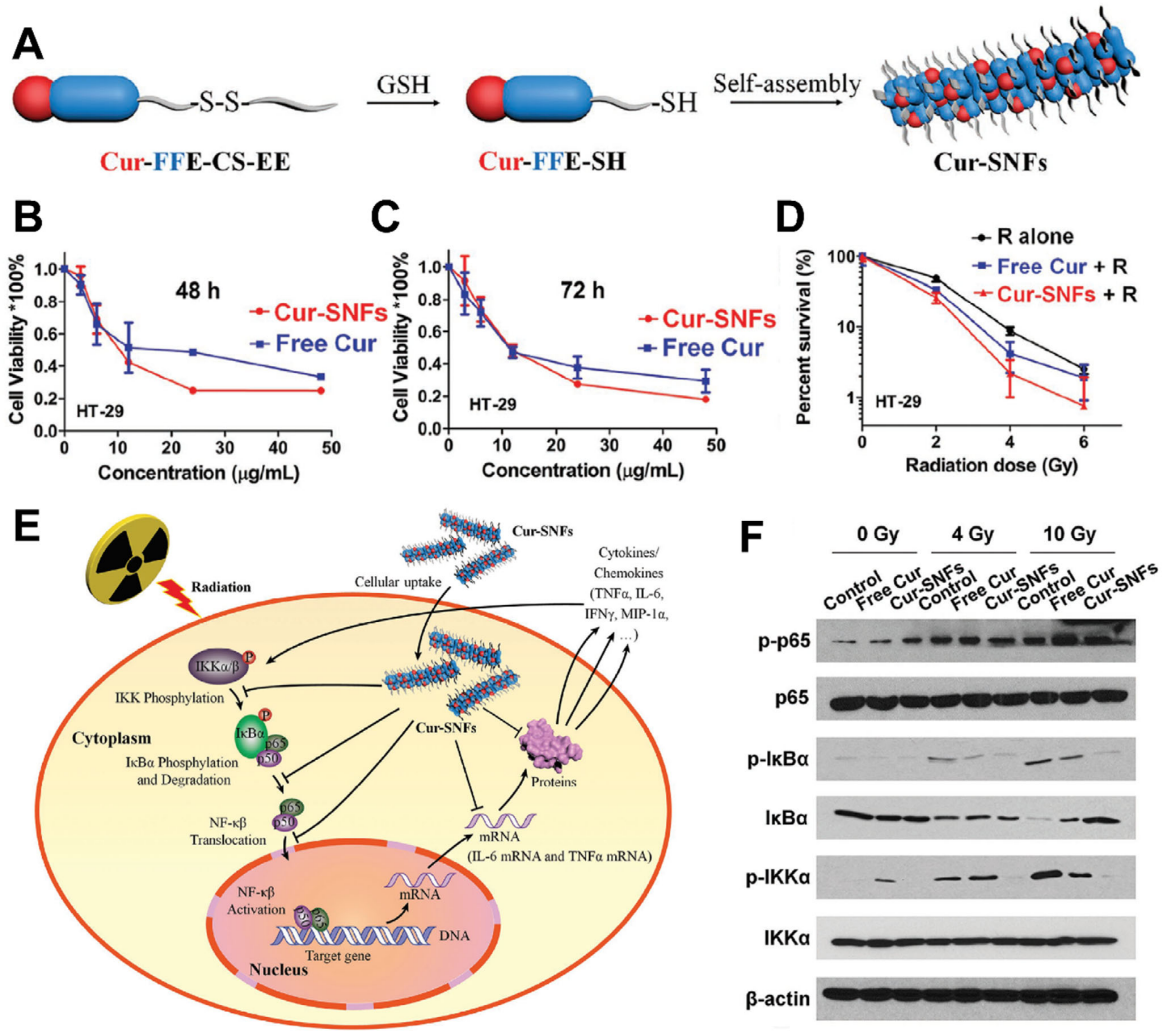
A) The formation of Cur‐SNFs triggered by GSH. In vitro cytotoxicities of Cur‐SNFs and Free Cur against HT‐29 cancer cells at B) 48 h and C) 72 h. D) The survival curve of HT‐29 cells after various treatments indicated by clonogenic assay. E) The molecular mechanisms of Cur‐SNFs. Reproduced with permission.^[^ ^123^ ^]^ Copyright 2018, Wiley‐VCH.

Redox reactions exhibit good flexibility in inducing the formation of peptide carriers.^[^ ^121^ ^]^ Curcumin, an active ingredient in the rhizomes of *Curcuma longa*, can be used as a radiosensitizer; however, its efficacy is not ideal.^[^ ^122^ ^]^ As shown in **Figure** **13**, Xu et al. designed a radiosensitizer (Cur‐SNFs) using a self‐assembled short peptide that could transform nanofibers into hydrogels to incorporate curcumin, triggered by reductants, such as GSH (Figure 13A).^[^ ^123^ ^]^ Cur‐SNFs exhibits a stronger radiation enhancement effect than free curcumin on colorectal cancer (Figure 13B,D). Moreover, mechanistic investigations showed that Cur‐SNFs effectively inhibit the NF‐κB pathway, thereby restoring the radiosensitivity of cancer cells (Figure 13E,F). Amphiphilic molecule precursors can consist of a hydrophilic part, a hydrophobic part, and a disulfide bond linking them. When the disulfide bond is cleaved by a reducing microenvironment, the hydrophobic part can be released and subsequently form assembly. In this case, a peptide of E3C16‐SS‐EIY that could self‐assemble into twisted or flat nanoribbons was reported. When GSH is reduced, E3C16SH and HSEIY are released from E3C16‐SS‐EIY to self‐assemble into short nanorods, resulting in a promising multi‐mechanism combination therapy for cancer. Several commonly used drugs, such as camptothecin,^[^ ^124^ ^]^ curcumin,^[^ ^125^ ^]^ and paclitaxel,^[^ ^126^ ^]^ have been conjugated into short peptides via disulfide bonds to realize GSH‐responsive drug release and a reduction in toxic side effects.

#### 3.2.4 Multiple‐Signal‐Induced Peptide Carriers

**Figure 14:**
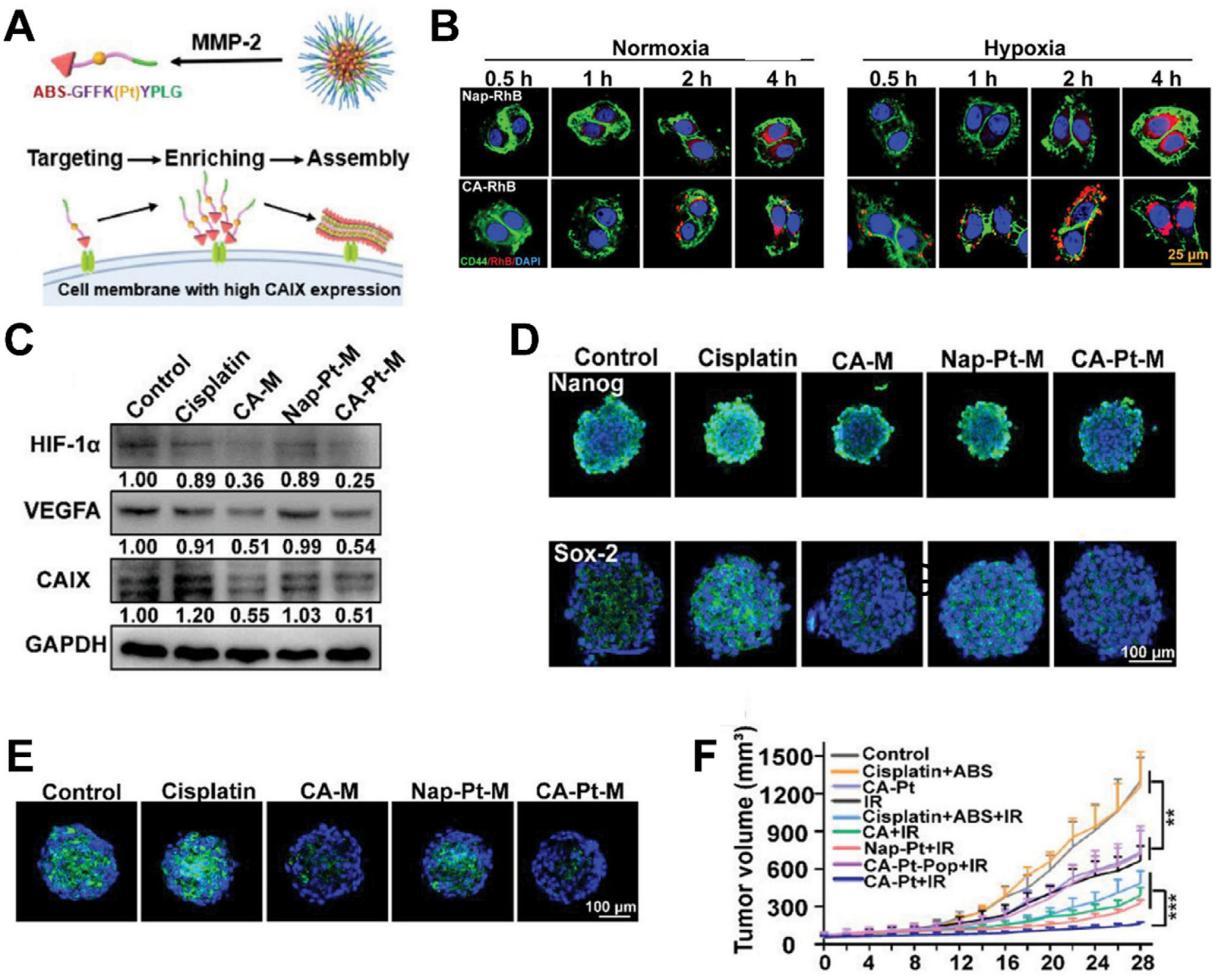
A) The formation process of CAIX‐induced surface assemblies. B) Fluorescence tracking of CA‐Pt and its control group in the cells cultured in normoxic and hypoxic conditions. C) The CAIX, VEGFA, and HIF‐1*α* expression after different treatments. D) The expression of Sox‐2 after different treatments. E) Laser Scanning Confocal Microscopy (LSCM) images of the hypoxic core after various treatments. F) Tumor volume growth curves during the therapy process. Reproduced with permission.^[^ ^128^ ^]^ Copyright 2023, Wiley‐VCH.

CSCs are known to be the origin of tumor radioresistance because of their indefinite self‐renewal and differentiation properties; however, targeting CSCs remains a great challenge.^[^ ^127^ ^]^ Liu et al. constructed a nanoprodrug (CA‐Pt) that targets hypoxic CSCs.^[^ ^128^ ^]^ As shown in **Figure** **14**A, CA‐Pt assembled into micelles in vitro and underwent sequential processes of “monomer release‐target accumulation‐surface self‐assembly” triggered by MMP‐2 response and carbonic anhydrase IX (CAIX, a marker for hypoxic CSCs) targeting. The capabilities of CA‐Pt to target and inhibit CAIX are shown in Figures 14B,C, respectively. CA‐Pt effectively reduces CSC stemness (Figure 14D) and reverses the hypoxic niche (Figure 14E). Finally, CA‐Pt treatment effectively assists RT in inhibiting tumor growth in mouse models of lung cancer (Figure 14F). Drug carriers with multiple signaling responses may be a developmental trend in the exploration of intelligent drugs.

### 3.3 Targeted‐Peptide‐Modified Radiosensitizers

Common radiosensitizing drugs without a defined target are typically distributed systemically, leading to increased damage to normal tissues outside the irradiated target zone.^[^ ^95^ ^]^ Therefore, maximizing drug enrichment at the lesion location as much as possible is necessary. The lesion location can serve as a port for radiosensitizers and targeted peptides can act as anchors that guide the radiosensitizers to the harbor.^[^ ^129^ ^]^ Targeted peptide modifications play an important role in the development of radiosensitizers.

MMPs are a family of zinc‐dependent endopeptidases that are over‐regulated in the TME and promote tumor invasion and metastasis.^[^ ^129^ ^]^ They can also be used as target enzymes for the design of radiosensitizer delivery systems. MMP‐responsive peptides can be designed as targeted peptides for tumors.^[^ ^130^ ^]^ For example, drug‐delivery platforms decorated with MMP‐2‐degradable peptides, such as Gly‐Pro‐Leu‐Gly‐Val‐Arg‐Gly‐Lys (GPLGVRGK), demonstrate vasculature‐specific targeting effects.^[^ ^131^ ^]^ Gao et al. constructed a peptide‐assembled nanostructure known as NIA‐LLPLGLAG‐D1, which included the MMP‐2‐responsive peptide PLGLAG, the radiosensitizer 2‐(2‐nitroimidazol‐1‐yl) acetic acid (NIA), and the PD‐L1 antagonist D1.^[^ ^132^ ^]^ With the help of PLGLAG, NIA‐LLPLGLAG‐D1 can be effectively enriched at tumor sites and selectively release NIA, ultimately improving tumor radiation sensitivity and activating the immunological system.

Moreover, targeting α_v_β_3_‐integrin also has applications in the development of radiosensitizers. AuNPs attached to the RGD peptide achieve site‐specific tumor accumulation, effectively improving the efficiency of RT.^[^ ^133^ ^]^ iRGD (CRGDK/RGPD/EC) is a tumor‐homing peptide that can effectively deliver therapeutic molecules to tumors.^[^ ^134^ ^]^ Neuropilin‐1 (NRP‐1) is overexpressed on the surface of blood vessels and head and neck squamous cell carcinoma tumor cell membranes and can be recognized by the CRGDK peptide.^[^ ^135^ ^]^ Xu et al. engineered a radiosensitizer by integrating the CRGDK peptide, which recognizes the target NRP‐1 to effectively deliver proteolysis‐targeting chimera prodrugs.^[^ ^136^ ^]^ This prodrug downregulates the BRD4‐RAD51AP1 pathway, providing a potential platform for tumor‐specific radiosensitization.

Bioinformatic analyses showed that the expression level of C‐C motif chemokine ligand 5 (CCL5) was significantly higher in patients with GBM than in healthy subjects; thus, it may be used as a target to guide drug delivery.^[^ ^137^ ^]^ Zhang et al. designed a CCL5 peptide and NO donor conjugate of a furoxans–gemcitabine (NG)‐modified bioinspired lipoprotein system (C‐LNG), in which the CCL5 peptide was used to target the GBM site and NG was used as a radiosensitizer to inhibit DNA repair activity.^[^ ^138^ ^]^ C‐LNG was found to preferentially target GBM tumor sites and significantly inhibit tumor growth in two orthotopic GBM tumor models.

**Figure 15:**
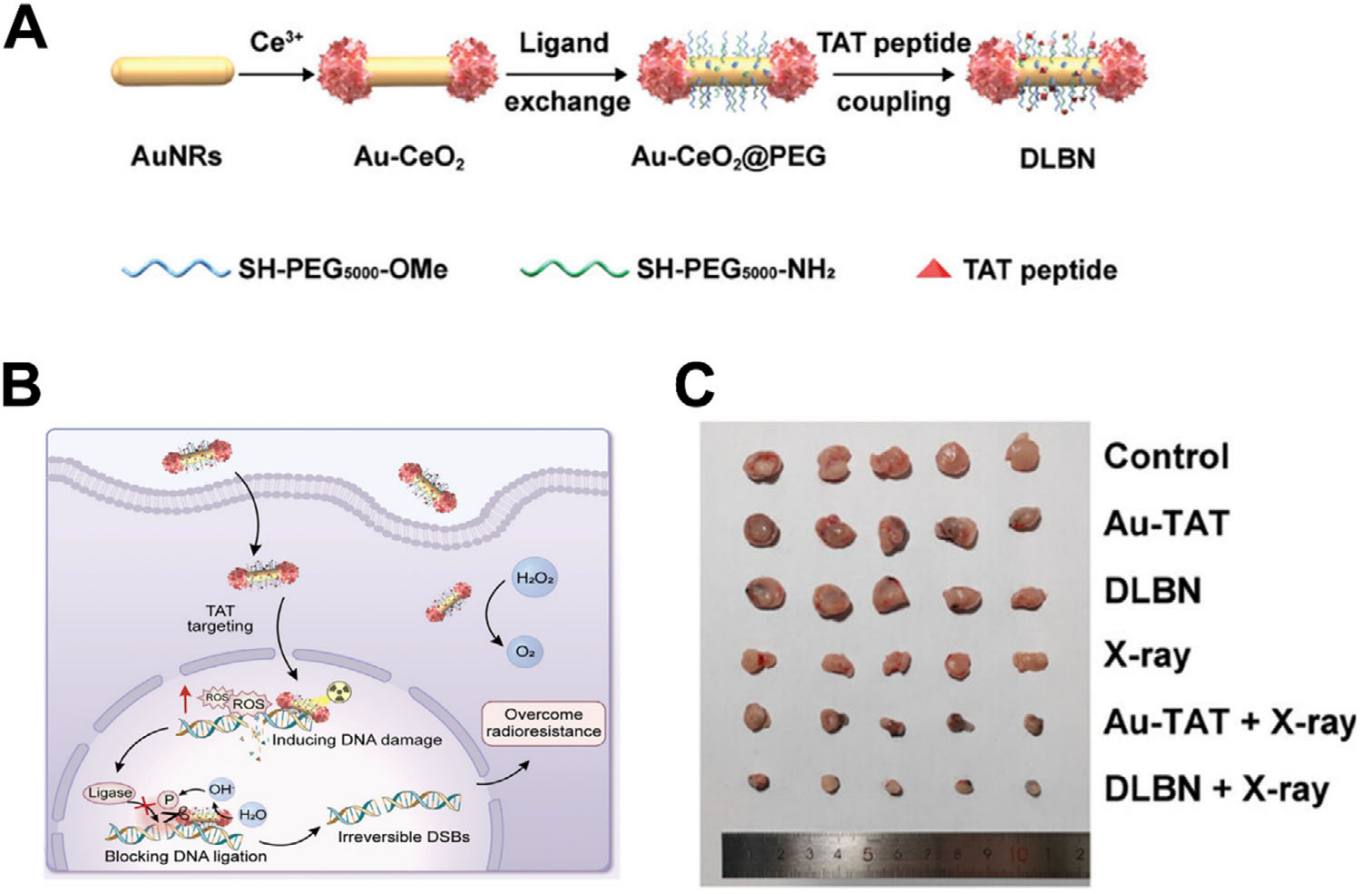
Schematic illustration of A) the design and synthesis of DLBN. B) the radiosensitization mechanisms of DLBN. C) Optical photographs of tumors collected from different groups. Reproduced with permission.^[^ ^141^ ^]^ Copyright 2024, Wiley‐VCH.

The sequence GRKKRRQRRRPQ, known as the TAT peptide, has both cellular penetration and nuclear‐targeting functions. Native proteins with surface‐modified TAT peptides have been used for effective nuclear‐targeted delivery.^[^ ^139^ ^]^ TAT is also used as a targeted peptide for the RT of tumors. Wang et al. prepared multifunctional peptide‐modified ultrasmall nanoparticles known as Au@Tat‐R‐EK NPs consisting of Tat‐targeting peptides to increase radiosensitizing efficacy.^[^ ^140^ ^]^ Ling et al. reported a nucleophilic radiosensitizer (DLBN) consisting of engineered Au nanorods and TAT peptides, which can induce irreversible DNA damage within cell nuclei by dephosphorylating DNA to effectively block DNA ligation (**Figure** **15**A).^[^ ^141^ ^]^ As shown in Figure 15B, DLBN directly attacks tumor cell nuclei as directed by the TAT peptide, with enhanced ROS generation and DNA damage upon X‐ray irradiation to overcome radioresistance. When incubated with fluorescently labeled DLBN, the signals of the Au‐TAT and DLBN groups were higher than those of the group without the TAT peptide, indicating nuclear targeting. Remarkably, the radiosensitization efficacy of DLBN in vivo under X‐ray irradiation was confirmed in nude mice bearing 4T1 tumors, based on significant tumor growth inhibition (Figure 15C).

## 4 Peptide‐Radionuclide Conjugates

**Figure 16:**
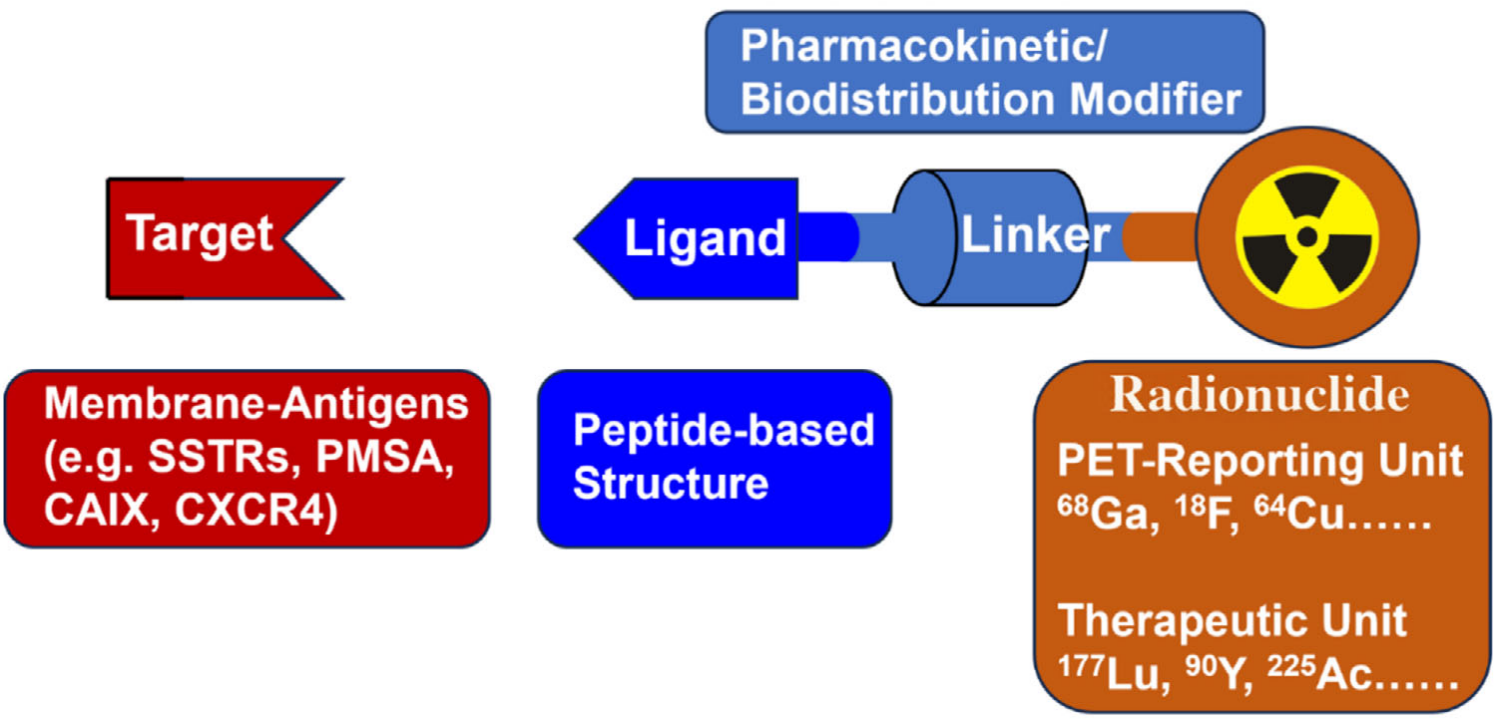
The composition of PRCs.

Peptide‐drug conjugates (PDCs) are emerging targeted therapeutic drugs that have attracted widespread attention in recent years.^[^ ^142^ ^]^ PDCs are mainly constructed from three components: target peptides, linkers, and payloads that exert cytotoxic or therapeutic effects.^[^ ^143^ ^]^ With the rapid development of proteomics, phage display, and SPPS technologies, an increasing number of new target peptides have been discovered or reasonably designed, promoting the development of PDCs.^[^ ^142^, ^143^ ^]^ Payloads can be divided into chemical, peptide, protein, and radioactive isotopes.^[^ ^144^ ^]^ When using radionuclides as the payloads, the PDCs form the PRCs, as we discuss in this section (**Figure** **16**).

PRCs can be used as diagnostic or therapeutic drugs, depending on whether the radionuclides have imaging ability (as emitters of either γ or positron radiation) or therapeutic ability (as emitters of either auger‐, α‐, or β‐ radiation). **Table** **2** lists the PRCs approved by the FDA. Motivated by PRCs currently on the market, there is a global wave of research on PRCs. Targeting peptides coupled with radionuclides can guide the precise targeting of radionuclides, mediate good tissue penetration and low immunogenicity, and ultimately achieve effective in vivo delivery. In this section, we review recent progress in PRCs with tumor therapy based on different targets.

**Table 2:** The PRCs approved for market by the FDA.

PRCs	Target	Tumor type	Aim	Company	References
[^177^Lu]Lu‐DOTATATE	SSTRs	Neuroendocrine tumors	Therapy	Novartis	[145]
[^111^In]In‐pentetreotide	SSTRs	Neuroendocrine tumors	Theranostic	Curium	[146]
[^68^Ga]Ga‐DOTATATE	SSTRs	Neuroendocrine tumors	Imaging	Novartis	[147]
[^68^Ga]Ga‐DOTATOC	SSTRs	Neuroendocrine tumors	Imaging	Uihc Pet Imaging	[147]
[^64^Cu]Cu‐DOTATATE	SSTRs	Neuroendocrine tumors	Imaging	RadioMedix	[148]
[^177^Lu]Lu‐PSMA‐617	PSMA	Prostate cancers	Therapy	Novartis	[149]
[^68^Ga]Ga‐PSMA‐11	PSMA	Prostate cancers	Imaging	UCLA	[150]

### 4.1 PRCs Targeting the Somatostatin Receptor

Neuroendocrine tumors (NETs) typically originate from peptidergic neurons and neuroendocrine cells, and approximately two‐thirds occur in the digestive system, including the stomach, intestines, and pancreas. Most NETs strongly express somatostatin receptors (SSTRs), which are G protein‐coupled receptors composed of seven transmembrane α‐helix structures and extracellular N‐terminal and tissue C‐terminal fragments. Intracellular and extracellular loops connect these structures, providing a basis for targeting using extracellular somatostatin and somatostatin analogs. Octapeptide octreotide was the first synthetic somatostatin analog approved by the FDA, followed by lanreotide and pasireotide. Benefitting from the high affinity of peptide derivatives derived from somatostatin for SSTRs, somatostatin analogs coupled with radioisotopes allow for the selective radioactive destruction of tumor cells overexpressing SSTRs. PRCs targeting SSTRs represent another advancing field in the therapy of NETs.

**Figure 17:**
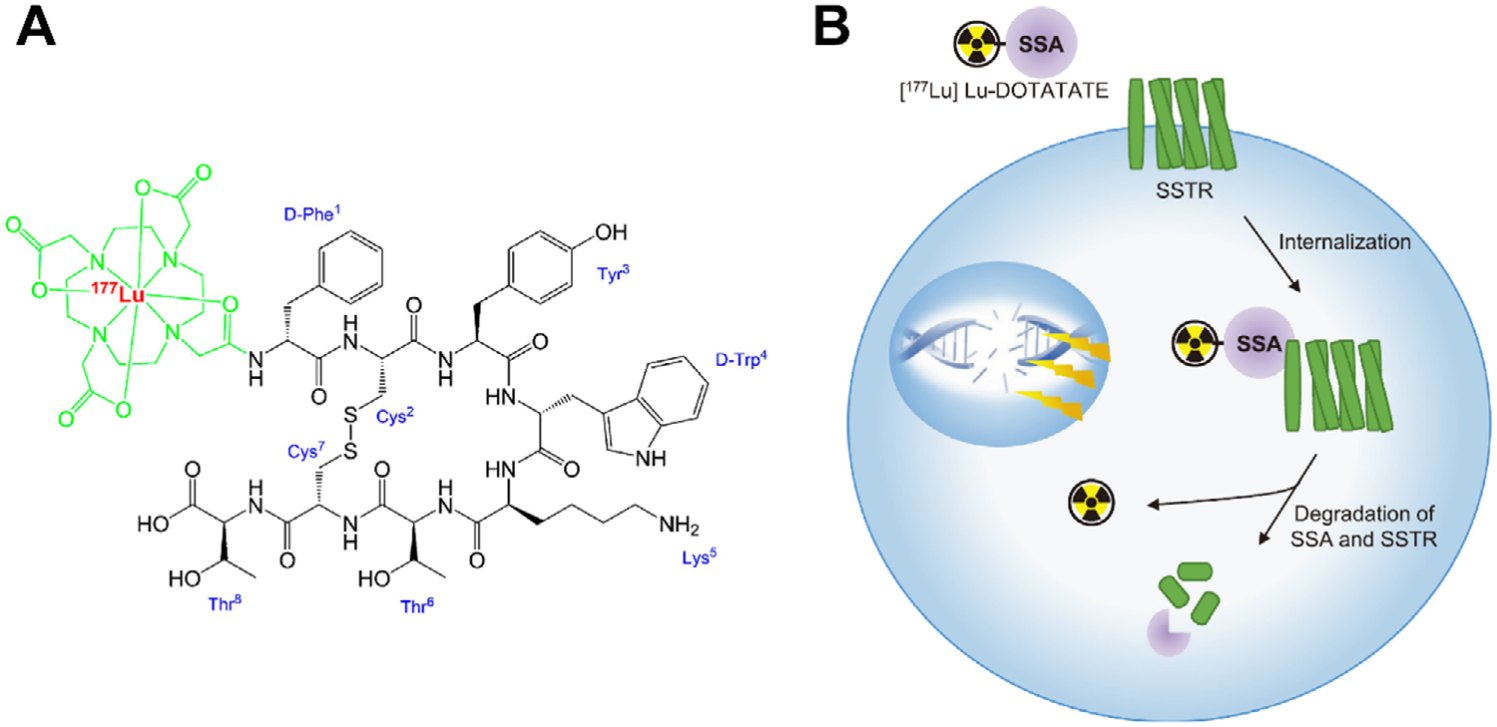
A) The structure of [^177^Lu]Lu‐DOTATATE. B) Mechanisms of action of [^177^Lu]Lu‐DOTATATE. Reproduced with permission.^[^ ^157a^ ^]^ Copyright 2022, Elsevier Inc.

^123^I‐modified octreotide ([^123^I]I‐octreotide) was the first PRC used for in vivo SSTR scintigraphy, performed by Krenning et al. in 1987.^[^ ^151^ ^]^ However, the cumbersome and expensive preparation of [^123^I]I‐octreotide and its nonspecific enrichment in the intestine led to the replacement of radioiodine with chelated ^111^In in 1989.^[^ ^151^ ^]^ [^111^In]In‐pentetreotide was obtained by covalently linking the diethylenetriaminepentaacetic acid (DTPA) group chelating ^111^In with the N‐terminal D‐Phe residue of octreotide,^[^ ^152^ ^]^ which was approved for SSTR‐positive tumor imaging by the FDA in 1994.^[^ ^153^ ^]^ Clinical studies on the use of [^111^In]In‐pentetrotide for tumor therapy were reported, but it exhibited some drawbacks, such as moderate efficacy and high costs.^[^ ^146^, ^154^ ^]^ The limited efficacy of [^111^In]In‐pentetreotide is related to the lack of intercalation of the electrons in the DNA helix. Therefore, more attention has been paid to different types of radionuclides.^[^ ^155^ ^] 90^Y and ^177^Lu are representative radioisotopes derived from β‐emitters. The better manageability of ^177^Lu‐labeled somatostatin analogs in metrology determines it as the compound of choice for PRCs.^[^ ^156^ ^]^ Octreotate has a high affinity for SSTRs, and the chelation of ^177^Lu with octreotate results in the formation of the therapeutic radiopharmaceutical ^177^Lu‐DOTA‐[Tyr^3^]‐octreotate ([^177^Lu]Lu‐DOTATATE, **Figure** **17**A). [^177^Lu]Lu‐DOTATATE binds to SSTR on the cell membrane, leading to the internalization of the drug by the cells. The accumulation of ^177^Lu within cells ultimately produces sufficient DNA double‐strand breaks after IR therapy to induce the apoptosis of cancer cells (Figure 17B). A randomized phase III NETTER‐1 trial conducted in 2012 and a study conducted in 2017 found that [^177^Lu]Lu‐DOTATATE improved disease‐free survival and had a favorable toxicity profile compared with nonreactive high‐dose octreotide in patients with SSTR‐positive gastroenteropancreatic NET.^[^ ^157^ ^]^ Finally, [^177^Lu]Lu‐DOTATATE, developed by the Novartis subsidiary Advanced Accelerator Applications, was approved for marketing by the FDA in 2018 for the treatment of gastric, intestinal, and pancreatic NETs.^[^ ^158^ ^]^

### 4.2 PRCs Targeting Prostate‐Specific Membrane Antigen

Prostate‐specific membrane antigen (PSMA), also known as folate hydrolase 1 (FOLH1), glutamate carboxypeptidase 2 (GCP2), and N‐acetylated‐alpha‐linked acidic dipeptidase I (NAALAD1), belongs to the peptidase M28 family and is highly expressed in prostate cancer.^[^ ^159^ ^]^ The extracellular structural domain of PSMA is sufficiently large, and its expression level in prostate cancer epithelial cells is 100–1000 times higher than that in normal tissue, making PSMA a classic molecular target for prostate cancer.^[^ ^159^ ^]^ Most importantly, recent studies have found that PSMA is overexpressed in the blood vessels of other solid tumors such as breast, gastric, and lung cancers, suggesting that PSMA is also an anti‐vascular therapeutic target for many tumors.^[^ ^160^ ^]^ Radiopharmaceuticals targeting PSMA can increase the concentration of radioisotopes at the tumor site and enhance their therapeutic effect, laying the foundation for the development of PSMA‐targeted therapeutic agents. Similarly, PRCs targeting PSMA have received considerable attention in recent years.

Through continuous screening and iterations, the most commonly used radioisotopes in radiopharmaceuticals targeting PSMA include ^68^Ga, ^131^I, ^177^Lu, and ^225^Ac.^[^ ^161^ ^]^ [^68^Ga]Ga‐PSMA‐11 is the first FDA‐approved PET imaging agent for prostate cancer, which combines the peptidomimetic Glu‐NH‐CO‐NH‐Lys(Ahx)‐HBED‐CC with the radionuclide ^68^Ga.^[^ ^150^, ^162^ ^]^ The specific recognition of PSMA by Glu‐NH‐CO‐NH‐Lys (Ahx)‐HBED‐CC allows for high intracellular accumulation, thus potentially detecting very small metastases.^[^ ^163^ ^]^ Moreover, the ^131^I‐labeled chemokine MIP‐1095 led to the first human therapy with PSMA ligands.^[^ ^161^ ^]^ PSMA‐617 is a small molecule peptidomimetic with faster plasma clearance, higher PSMA affinity, and lower toxicity.^[^ ^164^ ^]^ [^177^Lu]Lu‐PSMA‐617 (trade name Pluvicto) is a successful PRC targeting PSMA in tumor therapy. [^177^Lu]Lu‐PSMA‐617 consists of PSMA‐617 and a therapeutic radionuclide (^177^Lu), which can localize prostate cancer cell populations through PSMA‐617 and deliver radiation sources to prostate cancer cells, enabling precise radiation without affecting surrounding cells.^[^ ^165^ ^]^ Results from the global Phase III clinical trial showed that [^177^Lu]Lu‐PSMA‐617 significantly improved overall survival and radiographic progression‐free survival in patients with PSMA‐positive metastatic desmoplasia‐resistant prostate cancer, and reduced the risk of death in patients by 38% compared to the best standard of care.^[^ ^166^ ^]^ In addition to ^177^Lu, it can also be combined with a variety of radionuclides, including ^111^In and ^90^Y, for the diagnosis and treatment of prostate cancer.^[^ ^167^ ^]^

Since α‐rays have the advantages of high density and energy, a short range, a strong biological effect, a strong killing effect against tumor cells, and low toxicity to normal cells, many pharmaceutical companies are committed to developing targeted α‐particle therapy.^[^ ^168^ ^]^ Among the many α particles, ^225^Ac is a nuclide that has been widely researched for α‐radionuclide‐targeted therapy.^[^ ^169^ ^] 225^Ac‐labeled PSMA‐617 has shown therapeutic potential for the treatment of advanced prostate cancer.^[^ ^170^ ^]^ A clinical study showed that patients with multiple lesions throughout their body, as revealed by [^68^Ga]Ga‐PSMA‐11 PET/CT imaging, show substantial resolution of the lesions after two treatments with ^225^Ac‐labeled PSMA‐617.^[^ ^171^ ^]^ Moreover, several clinical trials have demonstrated that advanced metastatic castration‐resistant prostate cancer, which progressed despite treatment with [^177^Lu]Lu‐PSMA‐617, showed significant antitumor effects after treatment with ^225^Ac‐labeled PSMA‐617, which is an effective and tolerable treatment that improves patient survival.^[^ ^172^ ^]^

### 4.3 PRCs Targeting CAIX

CAIX is a cell surface glycoprotein overexpressed in various types of tumors.^[^ ^173^ ^]^ In low‐oxygen environments, the expression level of CAIX is significantly increased. CAIX plays a crucial role in maintaining the pH balance of tumor cells, helping them survive and proliferate in acidic and hypoxic environments.^[^ ^174^ ^]^ In a recent study, our group developed a CAIX‐targeted peptide derivative that overcame the radioresistance of cancer stem‐like cells.^[^ ^128^ ^]^ The functions and locations of CAIX provide a reliable target for the therapy of solid tumors.

**Figure 18:**
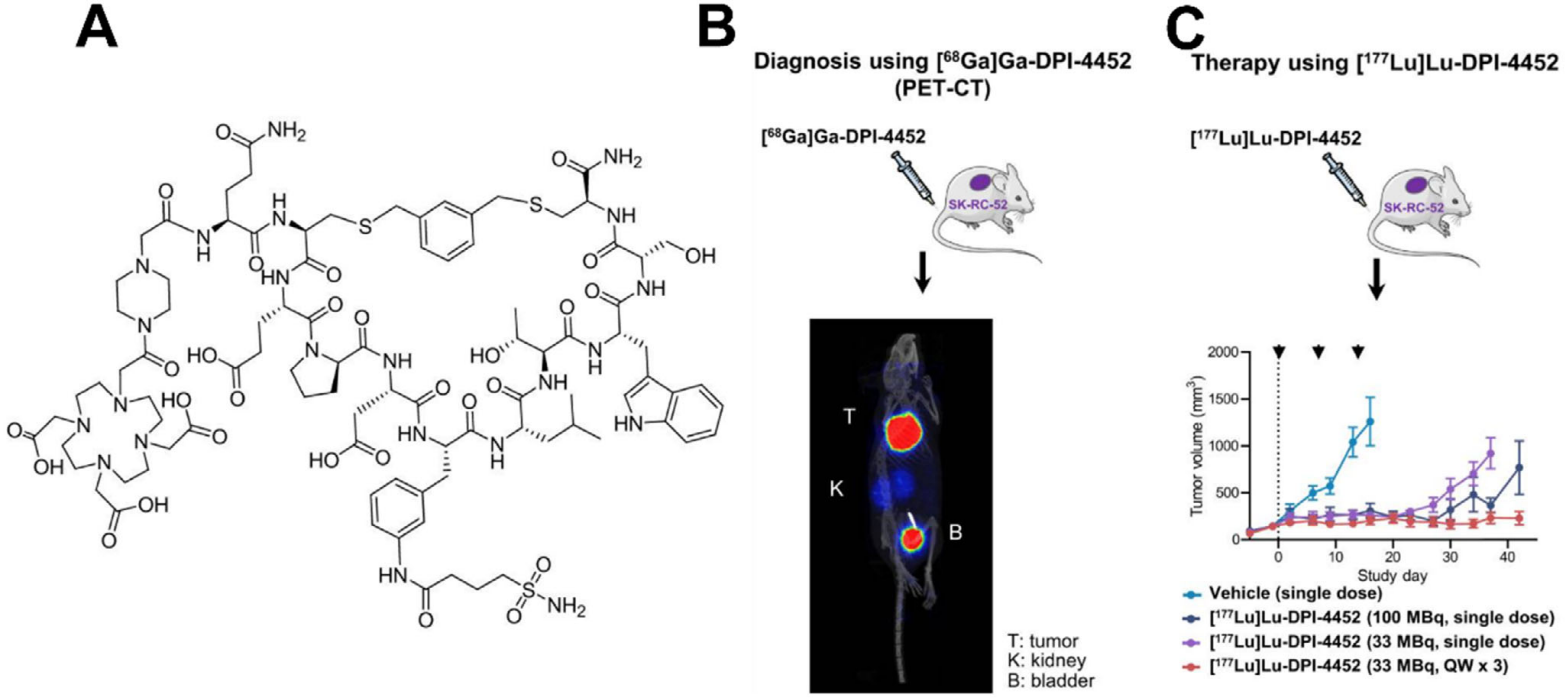
A) The structure of DPI‐4452. B) A PET/CT image of [^68^Ga]Ga‐DPI‐4452 in the mouse bearing a subcutaneous tumor. C) The tumor growth curve of tumor‐bearing mice treated with [^177^Lu]Lu‐DPI‐4452. Reproduced with permission.^[^ ^175^ ^]^ Copyright 2024, Society of Nuclear Medicine and Molecular Imaging.

[^68^Ga]Ga‐DPI‐4452 is one of the few currently reported PRCs targeting CAIX.^[^ ^175^ ^]^ Its ability to target CAIX depends on the CAIX‐targeting cyclic peptide DPI‐4452, and this cyclic peptide has a DOTA cage that chelates radionuclides for theranostics purposes (**Figure** **18**A). The results of the clinical study showed that [^68^Ga]Ga‐DPI‐4452 exhibited good imaging in patients with renal clear cell carcinoma. [^177^Lu]Lu‐DPI‐4452 also uses DPI‐4452 as the CAIX targeting component, demonstrating effective tumor therapeutic ability. The therapeutic effect of [^177^Lu]Lu‐DPI‐4452 can be evaluated via [^68^Ga]Ga‐DPI‐4452 (Figure 18B,C). The development of this radionuclide drug is expected to bring breakthroughs in the field of tumor therapy.^[^ ^175^ ^]^

### 4.4 PRCs Targeting CXC Chemokine Receptor 4

CXCR4 is a cell surface protein that plays important roles in the occurrence, chemotaxis, and metastasis of tumor cells.^[^ ^176^ ^]^ It is highly expressed in 75% of tumors (including pancreatic, breast, lung, prostate, colorectal cancers, and hematological malignancies) and is closely associated with tumor malignancy and patient prognosis.^[^ ^177^ ^]^ Moreover, it has been reported that CXCR4 not only plays a key role in tumors but is also strongly implicated in several immune disorders,^[^ ^178^ ^]^ the hereditary disease WHIM syndrome,^[^ ^179^ ^]^ and human immunodeficiency virus infections.^[^ ^180^ ^]^ Therefore, targeting CXCR4 may lead to new approaches in the development of drugs for multiple diseases.

The cyclic peptide cyclo(D‐Tyr^1^‐[NMe]‐D‐Orn^2^‐Arg^3^‐2‐Nal^4^‐Gly^5^)‐based probe [^68^Ga]Ga‐Pentixafor exhibits high affinity for CXCR4. As a molecular probe targeting CXCR4, it demonstrates good performance in imaging various advanced tumors.^[^ ^181^ ^]^ Encouraged by the effective imaging ability of [^68^Ga]Ga‐Pentixafor, therapeutic agents targeting CXCR4 based on cyclic peptides (cyclo(D‐3‐iodo‐Tyr^1^‐D‐[NMe]Orn^2^(AMBS‐(DOTA)‐Arg^3^‐Nal^4^‐Gly^5^) called ^177^Lu/^90^Y‐PentixaTher were further developed.^[^ ^182^ ^]^ Sarah M Jacobs et al. showed that there was a difference in the CXCR4 mRNA expression in glioblastomas. Therefore, glioblastomas that show positive via [^68^Ga]Ga‐Pentixafor might be further treated with [^177^Lu]Lu‐PentixaTher.^[^ ^183^ ^]^ There have been reported that [^177^Lu]Lu‐PentixaTher treatment is considered to be one of the promising therapeutic regimens for differentiated NET in G3 after the failure of first‐ and second‐line therapy,^[^ ^184^ ^]^ and encouraging outcomes were observed in a pilot trial evaluating the therapy efficacy of [^177^Lu/^90^Y]‐Pentixather for diffuse large B‐cell lymphoma.^[^ ^185^ ^]^ However, the largest clinical trials conducted so far have investigated [^177^Lu/^90^Y]‐Pentixather in advanced‐stage, heavily pretreated MM patients. Despite showing promising initial response rates, the treatment did not confer a meaningful overall survival benefit.^[^ ^182^ ^]^ Moreover, [^177^Lu/^90^Y]‐Pentixather can damage bone marrow and cause bone marrow ablation. This is practicable for haematological tumours as subsequent treatment with haematopoietic stem cell transplantation is possible. However, for solid tumors, this is a safety issue that needs to be further addressed, as patients with solid tumors are usually not able to regain their bone marrow function through stem cell transplantation in the same way that patients with haematological tumours do.^[^ ^186^ ^]^

### 4.5 PRCs Acting on Other Targets

FAP‐α is a type‐II transmembrane serine protease with a molecular weight of 97 kDa, which is highly expressed in breast and pancreatic cancer.^[^ ^187^ ^]^ Radiopharmaceuticals based on cyclic peptide of FAP‐2286, including [^68^Ga]Ga‐FAP‐2286, [^111^In]In‐FAP‐2286, and [^177^Lu]Lu‐FAP‐2286 can all image FAP‐positive tumors, and [^177^Lu]Lu‐FAP‐2286 also showed some anti‐tumor effects.^[^ ^67^ ^]^ Richard P. Baum's group recently presented first‐in‐human results using [^177^Lu]Lu‐FAP‐2286 and showed broad application for cancer, acceptable side effects, and a long retention time of the radiopeptide.^[^ ^188^ ^]^ Radiopharmaceuticals used in cancer theranostics primarily act on tumors and immune cells. In addition to the above targets, many radiopharmaceuticals in preclinical and clinical studies act on tumor‐cell‐related targets (e.g., gastrin‐releasing peptide receptor, HER2, urokinase‐type plasminogen activator receptor) and immune‐related targets (e.g., cluster of differentiation 8, 3, and 4).^[^ ^9b^ ^]^ Peptide ligands with high affinity for various targets are still in the stage of continuous development, and PRCs associated with these targets need to be further explored.

## 5 Conclusion and Outlook

In this review, the applications of peptide‐based biomaterials for RT are discussed in detail. In the preparation of radiosensitizers, peptide‐based biomaterials can be used either as a radiosensitizer, as a carrier of the radiosensitizing drug, or as a targeting peptide to guide the carrier to the tumor site. When used to construct radiopharmaceuticals, peptide‐based biomaterials serve as ligands that bind to targets. With the development of advanced technology, peptides with different amino acid sequences, special radiosensitizing activities, and high affinities for targets will be further developed, promoting the application of peptide‐based biomaterials in the field of RT.

However, peptide‐based biomaterials have certain drawbacks. Firstly, the in vivo stability of peptide‐based biomaterials is a noteworthy issue. Unstable amino acids in peptides may undergo isomerization,^[^ ^189^ ^]^ glycosylation,^[^ ^190^ ^]^ or oxidation,^[^ ^191^ ^]^ leading to the deterioration of the peptide‐based biomaterials. Moreover, the proteases existing in the human body can degrade peptide‐based biomaterials to influence their stability. Although cyclization,^[^ ^192^ ^]^ l‐configuration substitution,^[^ ^193^ ^]^ the use of other unnatural amino acids,^[^ ^194^ ^]^ and other peptidomimetic strategies have been applied to improve the stability of peptide‐based biomaterials, there is still a lack of a standard for evaluating their stability. Second, peptide‐based radiosensitizers are developed mostly at the preclinical research stage. They may have radiosensitizing effects at the animal level, but due to the differences between humans and experimental animals, their therapeutic effects in humans remain highly uncertain. This is particularly true for radiosensitizers based on in situ self‐assembled peptides, as there are currently no effective tools to monitor their self‐assembly behavior in vivo, and it remains unclear whether they function as intended. Finally, peptide‐based radiosensitizers or radiopharmaceuticals have insufficient targeting and delivery efficiency toward tumors. Targeting peptide modification enables tumor‐specific delivery, but its efficacy may be compromised due to tumor heterogeneity and variability in target expression. Meanwhile, to mediate the ideal targeting efficiency of peptide‐based radiosensitizers or radiopharmaceuticals on tumors, there is an urgent need to find new peptide ligands with high affinity for the targets.

Peptide‐based biomaterials have been widely applied in biology, such as for controlling cell fate, triggering biochemical signaling, and as peptide‐based vaccines, and have been rapidly developed for the preparation of radiosensitizers and radiopharmaceuticals. Despite some questions that remain to be answered, peptide‐based biomaterials have great potential as tools for developing innovative RT strategies and achieving clinical translation in the near future.

## Conflict of Interest

The authors declare no conflict of interest.
